# Bicalutamide and Trehalose Ameliorate Spinal and Bulbar Muscular Atrophy Pathology in Mice

**DOI:** 10.1007/s13311-023-01343-x

**Published:** 2023-01-30

**Authors:** Mariarita Galbiati, Marco Meroni, Marina Boido, Matilde Cescon, Paola Rusmini, Valeria Crippa, Riccardo Cristofani, Margherita Piccolella, Veronica Ferrari, Barbara Tedesco, Elena Casarotto, Marta Chierichetti, Marta Cozzi, Francesco Mina, Maria Elena Cicardi, Silvia Pedretti, Nico Mitro, Anna Caretto, Patrizia Risè, Angelo Sala, Andrew P. Lieberman, Paolo Bonaldo, Maria Pennuto, Alessandro Vercelli, Angelo Poletti

**Affiliations:** 1 Dipartimento di Scienze Farmacologiche e Biomolecolari “Rodolfo Paoletti”, Dipartimento di Eccellenza 2018-2027, Università degli Studi di Milano, Milan, Italy; 2 Department of Neuroscience Rita Levi Montalcini, Neuroscience Institute Cavalieri Ottolenghi, University of Turin, Orbassano, Italy; 3 Department of Molecular Medicine, University of Padova, Padua, Italy; 4 Unit of Medical Genetics and Neurogenetics, Fondazione IRCCS Istituto Neurologico Carlo Besta, Milan, Italy; 5 Department of Neuroscience, Vickie and Jack Farber Institute for Neuroscience, Weinberg ALS Center, Thomas Jefferson University, Philadelphia, PA USA; 6 Dipartimento di Scienze Farmaceutiche, Università degli Studi di Milano, Milan, Italy; 7 Department of Pathology, University of Michigan Medical School, Ann Arbor, MI USA; 8 Department of Biomedical Sciences, University of Padova, Padua, Italy; 9 Veneto Institute of Molecular Medicine (VIMM), Padua, Italy

**Keywords:** SBMA, Androgen receptor, Autophagy, Motor neuron, Skeletal muscle

## Abstract

Spinal and bulbar muscular atrophy (SBMA) is characterized by motor neuron (MN) degeneration that leads to slowly progressive muscle weakness. It is considered a neuromuscular disease since muscle has a primary role in disease onset and progression. SBMA is caused by a CAG triplet repeat expansion in the androgen receptor (*AR*) gene. The translated poly-glutamine (polyQ) tract confers a toxic gain of function to the mutant AR altering its folding, causing its aggregation into intracellular inclusions, and impairing the autophagic flux. In an in vitro SBMA neuronal model, we previously showed that the antiandrogen bicalutamide and trehalose, a natural disaccharide stimulating autophagy, block ARpolyQ activation, reduce its nuclear translocation and toxicity and facilitate the autophagic degradation of cytoplasmic AR aggregates. Here, in a knock-in SBMA mouse model (KI AR113Q), we show that bicalutamide and trehalose ameliorated SBMA pathology. Bicalutamide reversed the formation of the AR insoluble forms in KI AR113Q muscle, preventing autophagic flux blockage. We demonstrated that apoptosis is activated in KI AR113Q muscle, and that both compounds prevented its activation. We detected a decrease of mtDNA and an increase of OXPHOS enzymes, already at early symptomatic stages; these alterations were reverted by trehalose. Overall, bicalutamide and/or trehalose led to a partial recovery of muscle morphology and function, and improved SBMA mouse motor behavior, inducing an extension of their survival. Thus, bicalutamide and trehalose, by counteracting ARpolyQ toxicity in skeletal muscle, are valuable candidates for future clinical trials in SBMA patients.

**Supplementary Information:**

The online version contains supplementary material available at 10.1007/s13311-023-01343-x.

## Introduction

Spinal and bulbar muscular atrophy (SBMA), also known as Kennedy’s disease, is a rare X-linked neuromuscular disease for which there is no cure. SBMA is an adult-onset disease characterized by motor neuron (MN) degeneration in the brainstem and spinal cord anterior horns causing slowly progressive muscle weakness in hindlimb and bulbar muscles [1]. Muscle atrophy is due both to MN loss and to modifications occurring directly in skeletal muscle cells. Notably, myopathic changes often occur prior to MN degeneration in different SBMA mouse models [2–6].

The cause of SBMA is the expansion of a CAG triplet repeat in the first exon of the androgen receptor (*AR*) gene; the CAG stretch is translated into an elongated poly-glutamine (polyQ) tract in the N-terminus of the protein. In patients, the expanded CAG is longer than 38 contiguous triplet repeats, and the size of the expansion positively correlates with an earlier onset, but not with disease severity or progression rate [7]. AR is a ligand-activated transcription factor that mediates most of the androgenic activities in androgen-responsive cells in males [8, 9]. SBMA patients often present signs of androgen insensitivity (i.e., reduced fertility, erectile dysfunction, and gynecomastia) due to a partial loss of AR function caused by the elongated polyQ [10]. In addition, the polyQ tract confers a toxic gain of function to the mutant AR, which tends to misfold and aggregate, forming intracellular inclusions in the nucleus and cytoplasm of MNs and skeletal muscle fibers [11]. The role of AR inclusions in disease is still debated, but both their formation and toxicity depend on androgen binding [8, 12]. In fact, in absence of its ligand, AR (also in the elongated polyQ form) localizes in the cytoplasm complexed with chaperone proteins, and the polyQ tract is masked by the chaperones binding to the N-terminus. Upon ligand binding, chaperones are released, and the protein undergoes a conformational change that converts AR to an active status required for nuclear translocation and transcriptional regulation. Interestingly, several studies linked ARpolyQ toxicity to its nuclear localization induced by androgens [13–15]. Retention of misfolded AR in the cytoplasm correlates with an increased neuronal survival and an improvement of motor functions in SBMA mice [14, 15]. Androgen-induced ARpolyQ misfolding and intracellular aggregate formation also correlate with induction of autophagy, an exclusively cytoplasmic degradative system, which exerts protective functions in cells. Unfortunately, in SBMA affected cells the autophagic flux is blocked, thus preventing AR clearance [9, 16–19].

To date, several therapeutic strategies aimed either at diminishing androgen levels or at competing with androgens to block ARpolyQ activation and toxicity have been tested in mouse models of SBMA. Surgical castration ameliorates symptomatology in transgenic (Tg) SBMA mouse models [12], and chemical castration with leuprorelin [a gonadotropin-releasing hormone (GnRH) agonist that reduces testosterone release from the testis] rescues motor dysfunction and reduces ARpolyQ aggregation in SBMA mice [20, 21]. Unfortunately, leuprorelin in patients was less efficient than in mice [22], even if some benefits were proved [23]. Antiandrogens can prevent ARpolyQ misfolding by counteracting its activation [19, 24]. One of these is flutamide, a non-steroidal AR antagonist that was found beneficial in three SBMA mouse models [25]; instead, dutasteride, a 5alpha-reductase inhibitor that converts testosterone to a more potent androgen (dihydrotestosterone), did not modify disease progression in SBMA patients, even if some benefits were reported, such as a reduced tendency to fall [26].

In a neuronal cell model of SBMA, we demonstrated that bicalutamide, an FDA-approved type 1 pure antiandrogen, blocks the ARpolyQ activation by increasing its cytoplasmic degradation through autophagy process [27]. We also tested bicalutamide in combination with trehalose, a natural disaccharide with pro-autophagic activity and beneficial effects in neurodegenerative diseases [28–31], including SBMA [19, 27, 32]. By coupling the retention of ARpolyQ in the cytoplasm with its increased autophagic removal through the combined administration of bicalutamide and trehalose, we also demonstrated a synergistic effect of these two molecules in a motor neuronal SBMA model [27].

In this study, we utilized a knock-in (KI) SBMA mouse model (AR113Q) [33], which expresses AR under its endogenous promoter to better recapitulate the human disease, and we tested the effects of a single or combined administration of bicalutamide and trehalose. Our data demonstrate that bicalutamide extends the survival of AR113Q mice, and that both the compounds improve mouse motor behavior, and partially recover the morphology of muscle fibers. Bicalutamide, alone or in combination with trehalose, completely reverses the formation of AR insoluble species in the skeletal muscle of KI AR113Q mice, removing the autophagic flux blockage. Furthermore, we show that bicalutamide and trehalose prevent the activation of apoptosis, the decrease of mitochondrial DNA (mtDNA) and the accumulation of oxidative phosphorylation (OXPHOS) enzymes in muscle. Altogether, these results demonstrate that bicalutamide and trehalose represent a valuable therapeutic approach to counteract ARpolyQ toxicity.

## Methods

### Experimental Design

To analyze the effects of bicalutamide and trehalose on KI AR113Q SBMA mice, we administered: a) bicalutamide (Cayman Chemical, Ann Arbor, Michigan, USA, CAY14250) by subcutaneous injection (50 μL, 2 mg/Kg, dissolved in corn oil) twice a week; and/or b) trehalose (Bioblast-Pharma Ltd, Tel Aviv, Israel) in drinking water at 2% ad libitum, from 6 weeks of age until death or sacrifice (max 52 weeks). In a second experiment, animals were treated with bicalutamide from 12 weeks of age and sacrificed at 26 weeks of age. We analyzed: mouse survival curves, motor behavior (once a week), and molecular changes in the spinal cord and gastrocnemius muscle.

### Animals and Treatments

All the procedures involving animals and their care have been conducted according to the institutional guidelines, that follow Italian (D.L. no. 26/2014) and European laws and policies (2010/63/UE) on the protection of animals used for scientific purposes. All the experimental procedures were approved by The Ethics Committee of the University of Milan and by the Italian Ministry of Health (ref. 423/2015-PR). Mice were maintained at a temperature of 21 °C with 55 ± 10% relative humidity and a 12-h light/12-h dark cycle. Food (standard pellets) and water were supplied ad libitum.

KI-SBMA model (MGI: 88064) was originally developed by Lieberman’s laboratory [33]. In these mice, a portion of the coding region of mouse *Ar* exon 1 (Pro37 to Gly423) was exchanged for the same region in human *AR* exon 1, introducing a CAG repeat of 113 units. This SBMA mouse model maintains the physiological regulation of the *Ar* expression, being controlled by the endogenous promoter. However, since highly expanded CAG repeats are unstable and tend to decrease with continuous breeding, the female breeders of our colony carried a tract with 85 CAG repeats instead of 113; this mouse model is characterized by a less aggressive phenotype than that of the original colony, a delayed onset, and a highly variable disease progression. Despite this, the model recapitulates human disease features even better as the size of the polyQ tract is like the longest polyQ reported in SBMA patients (72 Qs) [34]. KI AR21Q mice (generated with the same approach but expressing a CAG repeat of 21 units) were used as control for the effects of humanizing mouse *Ar* gene. KI AR21Q mice are phenotypically similar to non-transgenic (NTg) ones [33].

Hemizygous female mice carrying AR113Q in the X chromosome were crossbred with C57Bl/6 J male mice to maintain the colony. A control strain of mice with 21 CAG repeats was also produced. The genotyping of the litters was conducted at weaning by PCR (Primer sequences, AR Forward: 5’-CCAGAATCTGTTCCAGAGCGTG-3’; AR Reverse: 5’-TGTTCCCCTGGACTCAGATG-3’) on DNA extracted from ear biopsies derived by identification procedure. Animals with substantial motor impairment had food on the bottom of the cage and water bottles with long drinking spouts. The humane endpoint was defined as the time when a mouse is unable to right itself within 30 s after being placed on one side or when it loses 20% of body weight. After genotyping, male mice were randomly assigned to different groups. A control group of animals received bicalutamide vehicle. The operator in charge of performing motor tests was blind for treatments (trehalose or water, and bicalutamide or oil), not for genotype (KI animals have an evident smaller size). Each mouse was weighed once a week starting at 6 weeks of age. NTg age-matched littermates were used as control mice. At the right stage (26 or 52 weeks of age), mice were deeply anesthetized with isoflurane (4%) and sacrificed by decapitation, spinal cords and gastrocnemius muscles were collected, snap-frozen, and maintained at − 80 °C or processed for muscle histological analysis.

### Motor Behavior Tests

Mice were brought to the testing room and allowed to acclimatize for 30 min. Motor coordination was measured by rotarod analysis (Ugo Basile Instruments, Gemonio, Varese, Italy). The test was performed weekly including two test trials at 30 rpm fixed speed for a maximum period of 600 s each, with a recovery time of 30 min between each test. The highest performance was used for analyses. Mice were trained two weeks before starting to register for the test.

For muscle force analysis, a grip strength meter (Ugo Basile Instruments) was used to measure forelimb grip strength. Mice were held by the tail and approached toward the apparatus. They were allowed to grasp the smooth metal horizontal pull bar with their forelimbs only and then were pulled backward until the applied force exceeded mouse strength to hold the bar. The peak tension (g) was recorded at this moment. Mice received a weekly session which included five consecutive trials (30 s of rest between trials); the highest and the lowest recording were discarded and the average of the other three was used to analyze muscle force production.

### Mass Spectrometry Analysis

#### Sample Preparation and Calibration Curves

Two hundred microliters of precipitation solution (i.e., acetonitrile added with 100 ng/mL internal standard, IS) were added to 50 μL of serum, or of muscle or spinal cord lysate [obtained by homogenizing 15 mg of tissue with a tissue lyser system (Qiagen, Hilden, Germany)]. Then, samples were centrifuged at 14,000 rpm for 10 min and the clear supernatant was injected onto the LC‐MS/MS system for analysis. The calibration curve was constructed by plotting the peak area ratio of compound/IS against the nominal concentration of compound standards in control mouse serum or tissue lysates. Following the evaluation of different weighting factors, the results were fitted to linear regression analysis with the use of a 1/x^2^ (x = concentration) weighting factor. The calibration curves had a correlation coefficient (*r*) of 0.99 or better.

#### HPLC Operating Conditions

A Shimadzu (Shimadzu, Kyoto, Japan) SIL series LC system equipped with degasser (DGU‐20A3), isopump (LC‐20AD) and column oven (CTO‐10AS) along with an auto‐sampler (SIL‐HTc) was used to inject 10 μL (for trehalose) or 2 μL (for bicalutamide) of the clear supernatant. For trehalose analysis an ACQUITY UPLC BEH AMIDE column (100 × 2.1 mm, 3.5 µm; Waters, Dublin, Ireland, UK) maintained at 35 ± 1 °C was used; an isocratic mobile phase comprising 0.1% ammonium hydroxide, and 0.1% ammonium hydroxide in acetonitrile (35:65, v/v) delivered at a flowrate of 0.25 mL/min was used for chromatographic resolution. For bicalutamide detection the aliquots were applied to an Atlantis dC18 column (50 × 4.6 mm, 3 µm; Waters), which was maintained at 40 ± 1 °C; an isocratic mobile phase comprising 0.2% formic acid/acetonitrile (35:65, v/v) delivered at a flowrate of 0.5 mL/min was used for chromatographic resolution.

#### Mass Spectrometry Operating Conditions

Quantitation was achieved by MS/MS detection in negative ion mode for bicalutamide, trehalose and IS using an MDS Sciex (Foster City, CA, USA) API‐4000 mass spectrometer, equipped with a Turboionspray™ interface at 500 °C. The common parameters, viz. curtain gas, GS1 gas and GS2 gas, were set at 20, 35 and 45 L/min, respectively, whereas the CAD gas was set at 6.0 L/min. The compound parameters, viz. de-clustering potential, enhanced potential, collision energy and collision exit potential, for bicalutamide (bicalutamide-d4, Santa Cruz Biotechnology, CA) and IS, were: − 60, − 11, − 20, − 5 V and − 62, − 7, − 25, − 4 V, respectively. Detection of the ions was performed in the multiple reaction monitoring (MRM) mode, monitoring the transition of the m/z 429.2 precursor ion to the m/z 255.2 product ion for bicalutamide and m/z 433.0 precursor ion to the m/z 255.1 product ion for IS; for trehalose the monitored transitions were m/z 341.1 for the precursor ion and m/z 179.1 119 m/z and 89 for product ions. Quadrupoles Q1 and Q3 were set on unit resolution. The analytical data were processed using Analyst software.

### Gastrocnemius Histological Analysis

Fresh gastrocnemius muscles (*n* = 3–5) were dissected, embedded in cryostat medium (Killik; Bio-Optica, Milan, Italy), frozen using cooled 2-methylbutane and cut on the cryostat (HM 550; Microm, Thermo Fisher Scientific, Waltham, MA, USA). Transverse 40 μm-thick sections were mounted onto 5% gelatin-coated slides. To evaluate muscle morphology, the sections were stained with hematoxylin/eosin (H&E; Merck, Darmstadt, Germania), dehydrated in ascending series of ethanol (95–100%) and cleared in xylene. Fiber perimeter, area, minimum (min) and maximum (max) Feret diameters were evaluated [35]: reconstructions and analysis of the sections were performed using a computer-assisted Nikon Eclipse E600 microscope equipped with the Neurolucida software (MicroBrightField, Williston, VT, USA) and the associated data analysis software NeuroExplorer (MicroBrightField).

### Stereological Counts of Lower MNs

Deeply anesthetized mice were transcardially perfused with 4% paraformaldehyde (PFA). PFA-fixed lumbar spinal cords (*n* = 3) were embedded in cryostat medium (Killik) and cut on the cryostat (HM 550; Microm) in transverse 50 μm-thick sections: free-floating sections were collected in an antifreeze solution and stored at − 20 °C. For Nissl staining, serial sections (1 every 400 μm) were washed in 0.1 M PBS (pH 7.4) and mounted on 4% gelatin-coated Superfrost slides (Bio-Optica); after air-drying, slices were hydrated in distilled water, then immersed in 0.1% Cresyl violet acetate (Merck, C5042) for 6 min, dehydrated in ascending series of ethanol (95–100%) and cleared in xylene. Finally, the slides were coverslipped with Eukitt (Bio-Optica). The stereological counts of MNs were performed along the tract L5–L6. Only the nucleoli of multipolar neurons with an area ≥ 200 μm^2^ (classified as alpha MNs) and located in the spinal ventral horns (Rexed lamina IX) were counted at 40 × , using the Optical Fractionator stereological technique, on a computer-assisted Nikon Eclipse E600 microscope equipped with the StereoInvestigator software (MicroBrightField) [36]. 5 µm guard zones, 100 × 100 µm counting frame size and 150 × 150 µm scan grid size were set up to perform the counts. The ventral horn volume of the considered segment and the number of the counted cells were obtained by NeuroExplorer Software (MicroBrightField). Quantification reports the number of MNs in L5-L6 sections.

### TUNEL Assay

Gastrocnemius muscles at 26 weeks of age were frozen in liquid nitrogen and cut into 40 μm-thick cross sections. Nuclei were stained with Hoechst 33,258 (Merck, B1155) and apoptotic nuclei were detected by the In Situ Cell Death Detection Kit, TMR red (Roche, Basel, Swisse, 12156792910), following manufacturer’s instructions. Images of the stained sections were captured randomly using a Zeiss LSM 700 laser-scanning confocal microscope equipped with a ZEN software (Carl Zeiss AG, Germany) and TUNEL-positive nuclei were quantified on the muscle area.

### RNA Extraction and RT-qPCR

Frozen tissues were homogenized with Tissue-Lyser II and stainless-steel glass beads (Qiagen) in TRI Reagent (Merck, T9424), and RNA was extracted following the manufacturer’s protocol. Total RNA was purified with Direct-zol RNA miniprep Plus kit (Zymo Research, Irvine, CA, USA, R2072), eluted in RNase-free water and quantified through Nanodrop (Thermo Fisher Scientific). Total RNA (0.5 µg) was reverse transcribed using the High-Capacity cDNA Reverse Transcription Kit (Thermo Fisher Scientific, 4368813) according to the manufacturer’s instructions.

Real-time PCR was performed using the CFX 96 Real-Time System (Bio-Rad Laboratories, Hercules, CA, USA) in a 10 µL total volume, using the iTaq SYBR Green Supermix (Bio-Rad Laboratories, 1725124). PCR cycling conditions were as follows: 94 °C for 10 min, 40 cycles at 94 °C for 15 s and 60 °C for 1 min. Primers for selected genes were designed using the program Primer 3 Plus and purchased from Eurofins Genomics (Ebersberg, Germany). They were used at a final concentration of 500 nM. Primer sequences are reported in Additional Table 1. Melting curve analysis was performed at the end of each PCR assay as a control for specificity. Data were normalized to the amount of the housekeeping gene ribosomal protein lateral stalk subunit P0 (*Rplp0*). Sample Ct values were used for target relative quantification through the ΔΔCt calculation. The N-fold changes in gene expression were obtained transforming data by the equation 2^−ΔΔCt^.

### Mitochondrial DNA Quantification

DNA was extracted from 25 mg of gastrocnemius muscle by NucleoSpin Tissue, Mini kit for DNA from cells and tissue (Macherey–Nagel, 740952) following manufacturer’s instructions. DNA content was quantified with NanoDrop (Thermo Fisher Scientific). 10 ng DNA were used for amplification with CFX 384 Real-Time System (Bio-Rad Laboratories) in a 10 µL total volume, using the iScriptTM One Step for Probes (Biorad, 1725141) and assessing mt-*CoxII* and *36B4* content as mitochondrial and nuclear-encoded genes, respectively. Primers and probes were obtained from Eurofins Genomics MWG Operon and are available upon request. The Ct values for each reaction were calculated and mtDNA content was determined as 2^−ΔΔCt^ fold difference of mtDNA from nuclear DNA (nDNA).

### Protein Extraction and Western Blot Analysis

To obtain total proteins, tissues were homogenized with Tissue-Lyser II and stainless-steel glass beads (Qiagen) in RIPA buffer (150 mM NaCl, 6 mM Na_2_HPO_4_, 4 mM NaH_2_PO_4_, 2 mM EDTA pH 8, 1% Na-deoxycholate, 0.5% Triton X-100, 0.1% sodium dodecyl sulfate) plus protease inhibitor cocktail (complete tablets, Roche Diagnostics GmbH, Mannheim, Germany, 04693116001) and phosphatase inhibitors (100 mM sodium orthovanadate and 100 mM sodium fluoride). Crude extracts were centrifuged for 10 min at 10,000 g at 4 °C to remove particulate matter. Supernatant protein concentration was determined by the bicinchoninic acid method (Cyanagen Reagents for Molecular Biology, Bologna, Italy, PRTD1,0500). Western immunoblot analysis was performed on 12% sodium dodecyl sulfate polyacrylamide gel electrophoresis loading 15 μg total proteins. Samples for OXPHOS reaction were not boiled before electrophoresis. Proteins resolved by electrophoresis were transferred to a 0.45 μm nitrocellulose membrane (AmershamTM ProtranTM Premium 0.45 μm NC, Cytiva, 10600003) through the Trans-Blot® Turbo™ transfer system (Bio-Rad Laboratories, 1704150). Nitrocellulose membranes were treated for 2 h with a blocking solution containing 5% non-fat dried milk (PanReac AppliChem ITW Reagents, Darmstadt, Germany, A0830,0500) in TBS-T [20 mM Trizma base, 140 mM NaCl (pH 7.6) and 0.1% Tween 20 (Merck, P1379)] and then incubated with the primary antibody overnight at 4 °C (anti-GAPDH, Immunological Science, MAB-10578, dilution 1: 5,000; anti-AR, Abcam, ab133273, dilution 1:5,000; anti-polyQ, Merck, P1874, dilution 1:3,000; anti-SQSTM1/p62, Merck, P0067, dilution 1:3,000; anti-HSPB8, R&D Systems, MAB4987, dilution 1:2,000; anti-BAG3, Abcam, ab47124, 1:3,000; anti-LC3, Merck, L8918, dilution 1:3,000; total OXPHOS Antibody Cocktail, Abcam, ab110413, dilution 1:3,000). Immunoreactivity was detected using the right secondary peroxidase-conjugated antibody: goat anti-rabbit (Jackson ImmunoResearch, 111–035-003, dilution 1:10,000) or goat anti-mouse (Jackson ImmunoResearch, 115–035-003, dilution 1:10,000). The immunoreactive regions were then visualized using the enhanced chemiluminescence detection kit reagents Westar Antares (Cyanagen, XLS0142). A ChemiDoc XRS+ System (Bio-Rad Laboratories, 1708265) was used for image acquisition. Optical intensity of the assayed samples was detected and analyzed using the Image Lab software version 5.2.1 (Bio-Rad Laboratories).

### Filter Retardation Assay

Total proteins (20 μg) were diluted in 100 μl of RIPA buffer and loaded in a dot-blot apparatus (Bio-Rad Laboratories, 1703938) to pass through a 0.22 μm cellulose acetate membrane (Whatman, 100404180). Proteins were fixed using 99% methanol and membranes were blocked for 2 h in 5% non-fat dried milk in TBS-T and incubated with anti-AR antibody (Abcam, ab110413, dilution 1:5,000) or anti-polyQ antibody (Merck, P1874, dilution 1:3,000) overnight. Membranes were rinsed with TBS-T, and incubated with the right secondary peroxidase-conjugated antibody. Signals were detected as for Western blot analysis.

### Testosterone Measurement

Testosterone levels in mouse serum were quantified through an ELISA kit (Fine Test, EM1850, Wuhan, China) following manufacturer’s instructions.

### Statistical Analysis

Comparisons between two groups were performed through two-tailed Student’s *t*-test (NTg vs. NTg plus trehalose) or one sample *t-*test (NTg vs. NTg plus bicalutamide). Mice survival was statistically evaluated through Log rank (Mantel-Cox test). Body weight, rotarod, and grip strength performances were analyzed by two-way analysis of variance (ANOVA) with time and genotype/treatment as factors, followed by Tukey’s test for multiple comparison. All the other analyses were done with a two-tailed Student’s *t*-test (NTg vs. KI AR113Q), followed by ANOVA with Tukey’s post hoc test to determine specific group pair(s) statistical difference among KI AR113Q groups. All the tests were performed with the PRISM software 8.0.2 (GraphPad, San Diego, CA, USA).

## Results

### Bicalutamide and Trehalose Improve Survival and Motor Behavior of KI AR113Q Mice

**Fig. 1:**
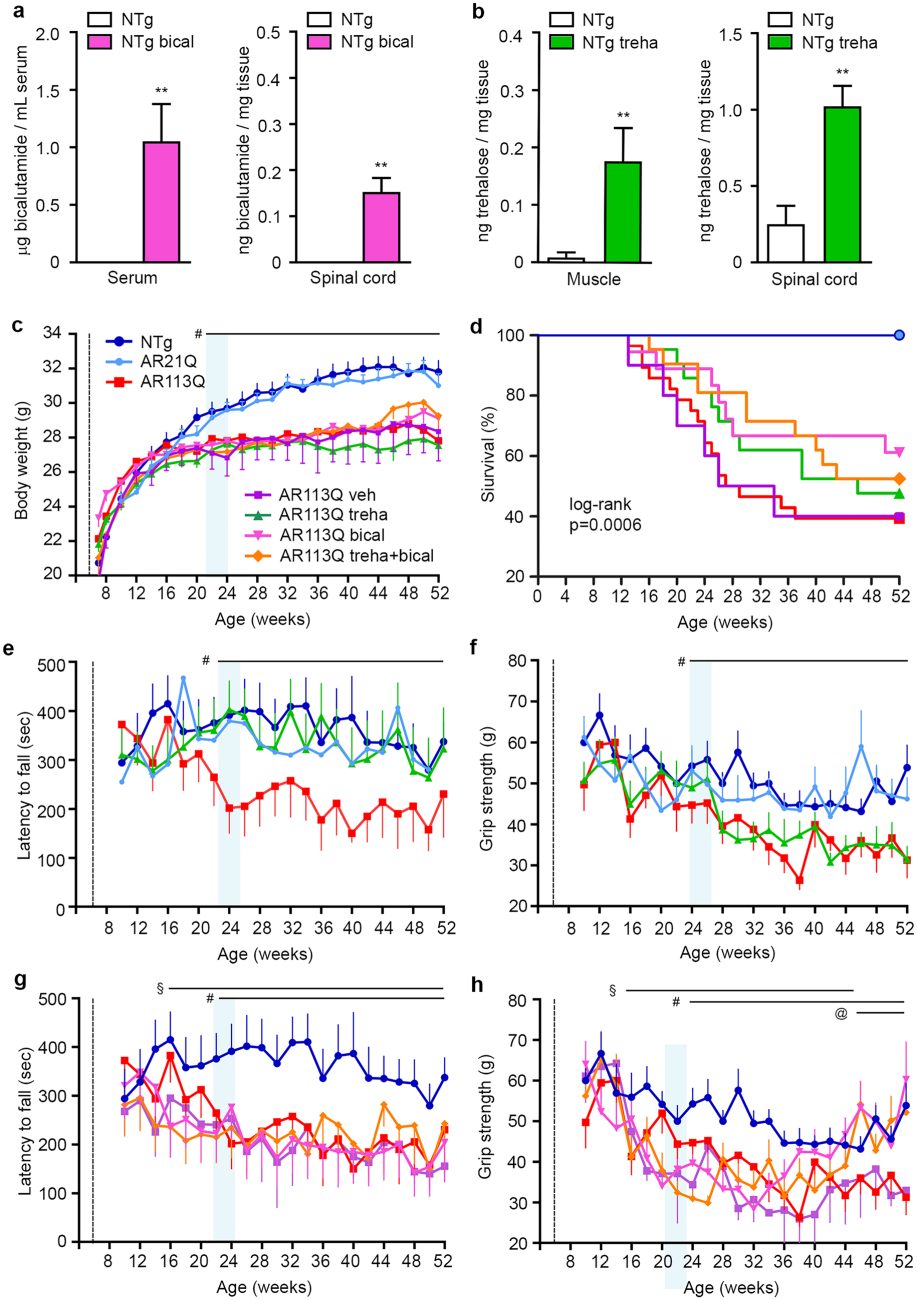
Bicalutamide and/or trehalose ameliorate SBMA KI mouse survival and motor behavior. LC–MS analysis of bicalutamide (**a**) and trehalose (**b**) levels in serum/spinal cord or muscle/spinal cord of non-transgenic mice (NTg, *n* = 4 per group) after 3 months of treatment. Bicalutamide was administered by subcutaneous injection (2 mg/Kg, dissolved in oil) twice a week; trehalose was administered at 2% in drinking water ad libitum. Two-tailed Student’s *t*-test for trehalose treatment (** *p* < 0.01), and one sample *t*-test for bicalutamide treatment. **c** Body weight analysis of NTg (blue, *n* = 13), KI AR21Q (light blue, *n* = 5), KI AR113Q mice (red, *n* = 28), and KI AR113Q mice treated with bicalutamide vehicle (veh, corn oil, purple, *n* = 5), trehalose (treha, green, *n* = 21), bicalutamide (bical, pink, *n* = 18) and trehalose plus bicalutamide (orange, *n* = 21). Treatments started at 6 weeks of age (dashed line) and continued until the sacrifice at 52 weeks of age. Data are expressed as mean ± SEM. The light blue vertical rectangle indicates the onset of the disease, i.e., when the body weight of the KI AR113Q mice starts to be statistically different *vs.* NTg mice (two-way ANOVA with time and genotype/treatment as factors, followed by Tukey’s test for multiple comparison) # *p* < 0.05. **d** Kaplan–Meier analysis of survival of NTg, KI AR21Q, KI AR113Q mice and KI AR113Q mice treated with bicalutamide vehicle, or trehalose, or bicalutamide, or trehalose plus bicalutamide. Log rank (Mantel-Cox test). *p* = 0.0006 *vs.* AR113Q. **e** Rotarod analysis of motor coordination and **f** grip strength test for forelimb force in NTg, KI AR21Q, KI AR113Q, and KI AR113Q trehalose-treated mice. The light blue vertical rectangle indicates the onset of the disease, i.e., when the latency to fall or the grip strength of the KI AR113Q mice starts to be statistically different *vs.* NTg mice (two-way ANOVA with time and genotype/treatment as factors, followed by Tukey’s test for multiple comparison) #*p* < 0.05. Data are expressed as mean ± SEM. **g** Rotarod analysis of motor coordination and **h** grip strength test for forelimb force in NTg, KI AR113Q mice and in KI AR113Q mice treated with bicalutamide or bicalutamide plus trehalose. The light blue vertical rectangle indicates the onset of the disease, i.e., when the latency to fall or the grip strength of the KI AR113Q mice starts to be statistically different from that of NTg mice (two-way ANOVA with time and genotype/treatment as factors, followed by Tukey’s test for multiple comparison) #*p* < 0.05, §, AR113Q bical or AR113Q treha + bical *vs.* NTg, *p* < 0.05; @, AR113Q bical or AR113Q treha + bical *vs.* AR113Q, *p* < 0.05

To assess the efficacy of bicalutamide and trehalose on KI AR113Q mice, they were treated with the two compounds alone or in combination. Male KI AR113Q mice (*n* = 18–28/group) were treated with bicalutamide (2 mg/kg, subcutaneously, twice a week) and/or 2% trehalose in drinking water starting at 6 weeks of age. The efficacy of the administration routes of the two compounds was tested by liquid chromatography–mass spectrometry (LC–MS) on skeletal muscle and spinal cord of treated NTg mice (Fig. 1a, b). As already reported by other authors [37], bicalutamide levels in the CNS are much lower than in plasma since this antiandrogen has a low crossing rate of the blood brain barrier (BBB). LC–MS analysis confirmed the presence of trehalose in both selected tissues, reaching levels similar to that previously reported [38]. Due to technical reasons (interference of many other sugars), we could not measure trehalose levels in mouse plasma. We analyzed KI AR113Q mouse body weight. Age-matched NTg or KI AR21Q male mice were used as controls. KI AR113Q mouse body weight was significantly different from that of NTg and KI AR21Q mice starting from 22 weeks of age (Fig. 1c). Bicalutamide and trehalose did not affect KI AR113Q mouse body weight up to 52 weeks of age. Treatments did not change the body weight of NTg mouse (Supplementary Fig. 1a).

KI AR113Q mouse survival analyzed by log-rank (Mantel-Cox) test was significantly different (*p* = 0.0006) from that of NTg mice, with a median survival of 28 weeks (Fig. 1d). Only 39% of KI AR113Q mice had survived to be 52 weeks of age. Trehalose increased the median survival to 46 weeks, and 48% of mice survived, even if the two survival curves were not found to be statistically different. The median survival of mice treated with bicalutamide either alone or in combination with trehalose exceeded the 52 weeks, with a survival of 61 and 52%, respectively, and this prevented us from performing additional statistical analysis aimed at evaluating the exact median survival in these groups. KI AR21Q mice survival was similar to that of NTg mice (Fig. 1d). A group of KI mice (n = 6) was treated with bicalutamide vehicle (oil), and their body weight and survival were not different from that of KI AR113Q animals (Fig. 1c, d). There were no considerable differences in the survival of treated NTg mice (Supplementary Fig. 1b); two NTg animals treated with bicalutamide were euthanized due to fight wounds. Since mouse enterocytes express trehalase that hydrolyzes trehalose into two molecules of D-glucose [39], we tested mouse glycemia levels that remained unchanged at sacrifice (data not shown).

**Fig. 2:**
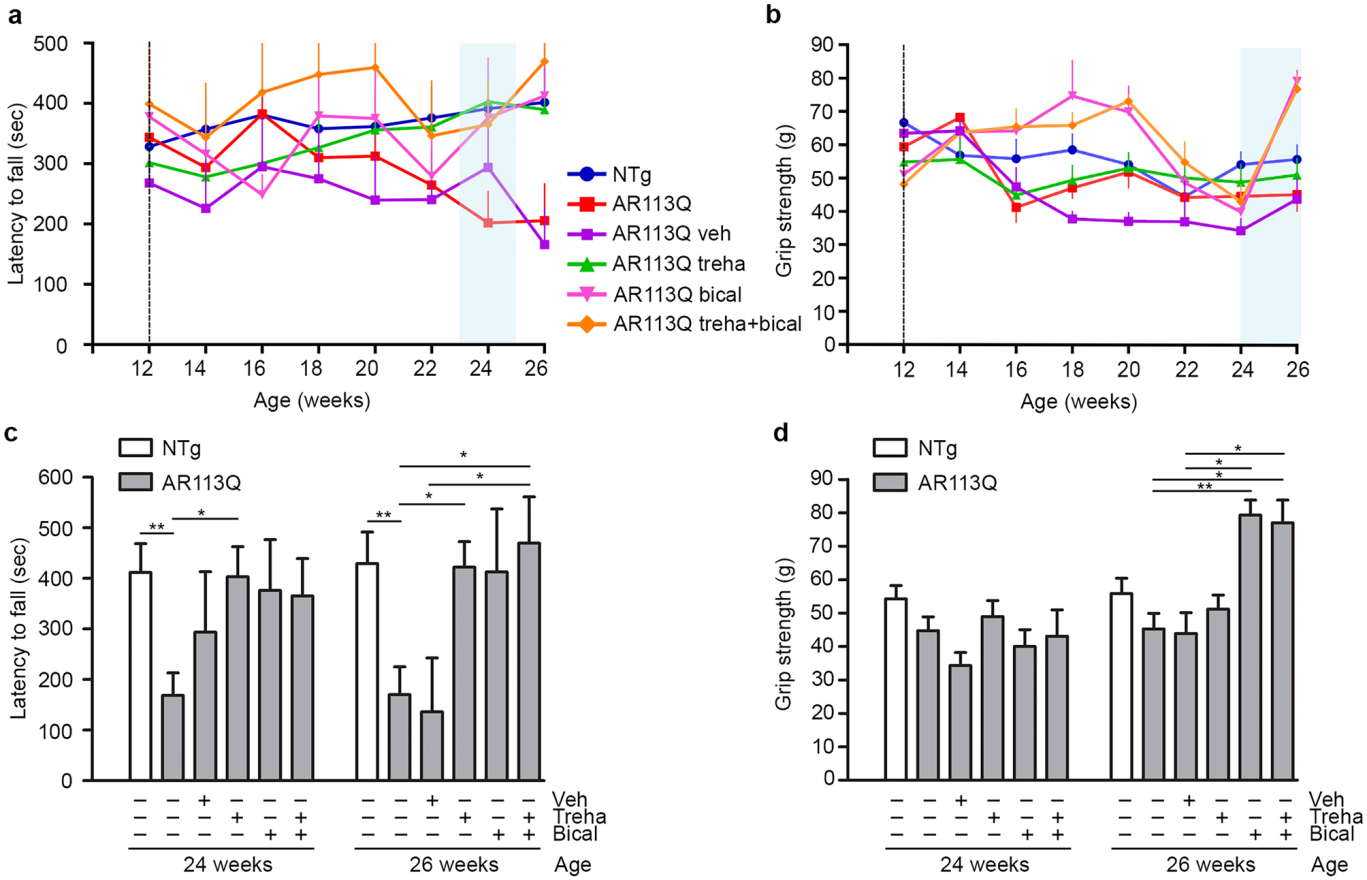
Effect of delayed bicalutamide treatment on SBMA KI mouse motor behavior. **a** Rotarod analysis of motor coordination and **b** grip strength test for forelimb force in NTg (blue, *n* = 13), KI AR113Q mice (red, *n* = 28), and KI AR113Q mice treated with bicalutamide vehicle (veh, corn oil, purple, *n* = 5), trehalose (treha, green, *n* = 21), bicalutamide (bical, pink, *n* = 5) and trehalose plus bicalutamide (orange, *n* = 5). The light blue vertical rectangle indicates the onset of the disease, i.e., when the latency to fall or the grip strength of the KI AR113Q mice starts to be statistically different from that of NTg mice (two-way ANOVA with time and genotype/treatment as factors, followed by Tukey’s test for multiple comparison). Trehalose treatment started at 6 weeks of age, while bicalutamide treatment started at 12 weeks of age (dashed line). Data are expressed as mean ± SEM. Histograms in **c** and **d** represent the mean ± SEM of latency to fall and grip strength (respectively) at 26 weeks of age in NTg and KI AR113Q mice. Two-tailed Student’s *t*-test between NTg and untreated KI AR113Q mice); ANOVA with Tukey’s post hoc test among KI AR113Q mouse groups. *p < 0.05; **p < 0.01

KI AR113Q mice had impaired motor performances starting from 24 weeks of age at rotarod test and from 26 weeks at the grip strength test (vertical light blue rectangle in Fig. 1e, f). Trehalose-treated mice fully recovered from low motor performance at the rotarod test (Fig. 1e), but not at the grip strength test (Fig. 1f). Bicalutamide increased the anterior paw force of KI AR113Q animals from 46 weeks of age (Fig. 1h). Motor performances and grip strength of vehicle-treated KI mice were not different from that of KI AR113Q animals (Fig. 1g, h); NTg mice motor behavior was not affected by treatments (Supplementary Fig. 1c, d). Bicalutamide-treated animals displayed a worsening of rotarod and grip strength performance earlier than KI AR113Q control mice, at 16 and 18 weeks, respectively (Fig. 1g, h). We postulated that this adverse effect of bicalutamide is probably due to its anti-anabolic activity on skeletal muscle, since we started our treatment at 6 weeks of age, when skeletal muscle development is not completed in mice. Given the fact that at this stage the anabolic activity of androgens supports muscle development in males, it is possible that the antiandrogen bicalutamide interferes with the late postnatal muscle developmental phase [40]. To avoid this adverse effect of bicalutamide, we repeated the study by postponing the beginning of the treatment to 12 weeks of age and evaluating its effect till the early symptomatic stage of the disease (26 weeks). In these new experimental conditions, both bicalutamide and trehalose completely prevented disease manifestation at 26 weeks, being the latency to fall values of these groups similar to those of NTg mice (Fig. 2a, c). Bicalutamide alone and in combination with trehalose was also able to recover anterior paw force in KI AR113Q mice (Fig. 2b, d).

This first set of data indicates that bicalutamide extends the survival of KI AR113Q mice and that both trehalose and bicalutamide are functionally associated with improved motor performance. Furthermore, results revealed a detrimental action of bicalutamide, but only if administered in very young mice, while no adverse effects were noted in older animals.

### Bicalutamide and Trehalose Counteract Morphological and Molecular Changes of KI AR113Q Mouse Muscle

**Fig. 3:**
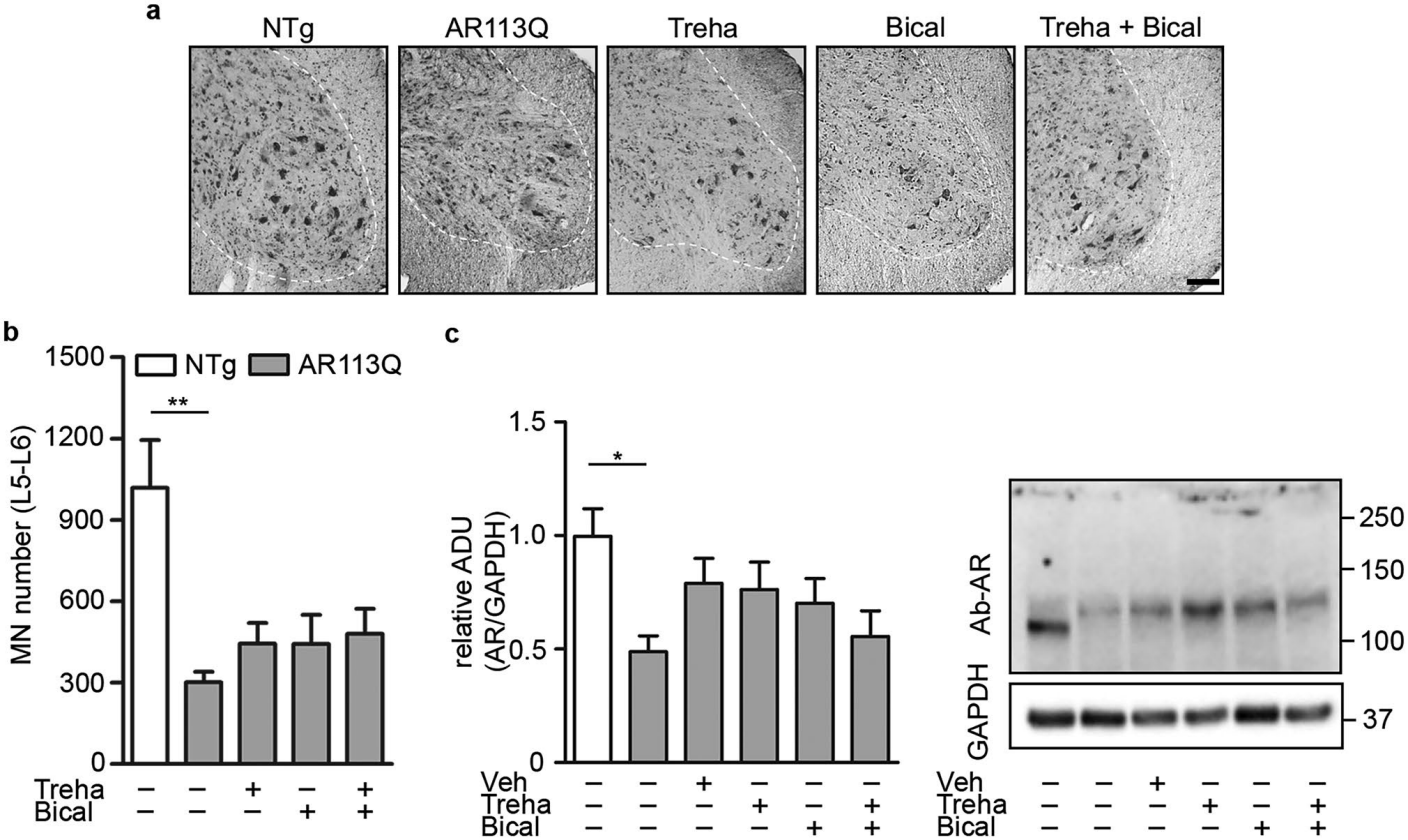
Histological and biochemical analysis of SBMA KI mouse spinal cord after bicalutamide and/or trehalose treatment. **a** Representative photomicrographs of Nissl staining of lumbar spinal cord ventral horns (L5–L6) of non-transgenic (NTg), KI AR113Q and treated KI AR113Q mice at 26 weeks of age. Scale bar = 50 μm. **b** Quantification of the somatic MN number in the ventral horns. Data are expressed as mean ± SEM, *n* = 3. Two-tailed Student’s *t*-test between NTg and KI AR113Q mice; **p < 0.01. ANOVA with Tukey’s post hoc test among KI AR113Q mouse groups (NS). **c** Western blot analysis reporting AR expression in the spinal cord of NTg, KI AR113Q and treated KI AR113Q mice at 26 weeks of age. AR levels were normalized using GAPDH as loading control. ADU, arbitrary densitometric units. Each bar represents the mean ± SEM of four independent replicates. Side panel shows a representative immunoblot. Two-tailed Student’s *t*-test between NTg and KI AR113Q mice. ANOVA with Tukey’s post hoc test among KI AR113Q mouse groups. *p < 0.05

SBMA was primarily considered a MN disorder, with ARpolyQ able to exert its toxicity in the bulbar region of the brainstem and the anterior horn of the spinal cord; currently, it has been reconsidered as a neuromuscular disease, since also muscle has a primary role in disease onset and progression [3, 6, 41, 42]. We initially wanted to evaluate if MN alterations occur in our animal model and to quantify AR expression in the spinal cord. From the morphological analysis reported in Fig. 3a and quantified in Fig. 3b, the somatic MN loss is evident in the anterior horn of the spinal cord (L5-L6 segment) of KI AR113Q mice already at 26 weeks of age. KI mice compared to age-matched NTg ones displayed a significant reduction of somatic MNs that is in line with the reduced motor performance. When we analyzed AR levels in the spinal cord, we found that the mutant monomeric ARpolyQ levels were reduced in KI AR113Q mice compared to NTg mice (Fig. 3c). Since in the spinal cord AR expression is mainly confined to the anterior horn MNs, this reduction of AR levels correlates with the reduced number of MNs found in morphological studies. Furthermore, the decreased levels of the AR protein are in line with previous evidence [33]. The levels of the monomeric AR were only partially restored by treatments (Fig. 3c). We also evaluated the presence of AR aggregates by filter retardation assay (FRA). The levels of AR aggregates were very low at 26 weeks of age and comparable to that of NTg mice (data not shown). Furthermore, either in basal conditions or in treated animals, we did not find any modification of the autophagic response in the spinal cords of KI AR113Q mice at 26 weeks of age both at the protein and mRNA levels (Supplementary Figs. 2a–d and 3a–e).

This set of data seems to exclude that the beneficial effects of bicalutamide and trehalose on mouse motor behavior are exerted at the central nervous system level, at least in the spinal cord, which is a target of ARpolyQ toxicity.

**Fig. 4:**
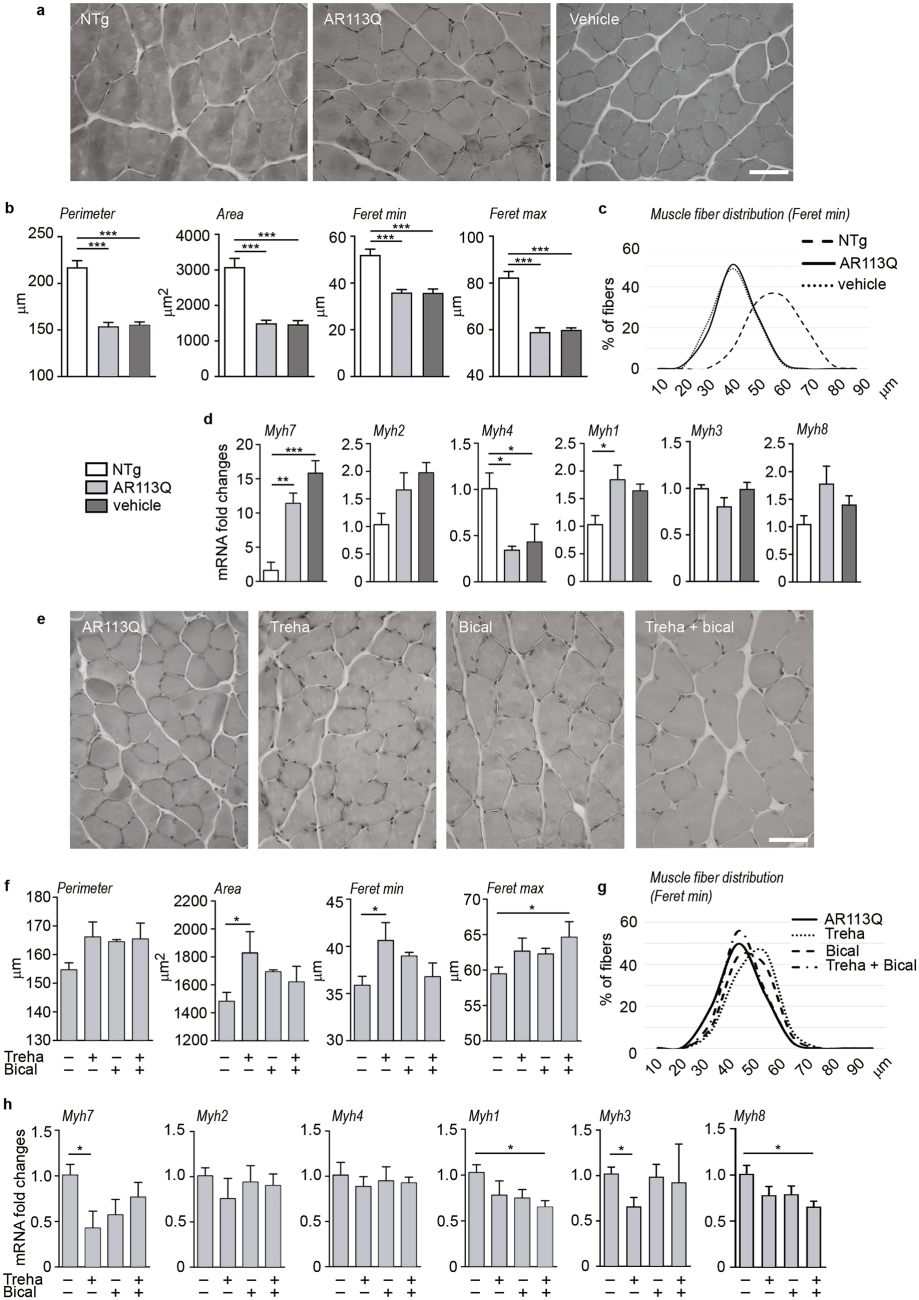
Histological and biochemical analysis of SBMA KI mouse gastrocnemius muscle after bicalutamide and/or trehalose treatment. **a** H&E staining reveals muscle pathology in the gastrocnemius muscle of SBMA mice at 26 weeks of age. Scale bar 50 μm. NTg, non-transgenic; vehicle, KI AR113Q treated with corn oil (bicalutamide vehicle). *n* = 4. **b** Analysis of gastrocnemius muscle fiber parameters (perimeter, area, minimum and maximum Feret diameters) in NTg (white columns), KI AR113Q (light grey columns) and vehicle-treated KI AR113Q mice (grey columns). Each bar represents the mean ± SEM of four independent replicates. ***p < 0.001; ANOVA with Tukey’s post hoc test. **c** Relative frequency distribution of myofiber min Feret diameter size sorted in NTg, KI AR113Q and vehicle-treated KI AR113Q mice. **d** Expression of genes coding for different myosin heavy chain isoforms in SBMA mouse gastrocnemius muscles. RT-qPCRs were performed on total RNA of NTg, KI AR113Q and vehicle-treated KI AR113Q mice at 26 weeks of age. Data were normalized to the amount of *Rplp0* mRNA, expressed relative to the levels determined in NTg mice taken as internal reference, and expressed as fold changes. Data are means ± SEM of 3/5 independent replicates. ANOVA with Tukey’s post hoc test among KI AR113Q mouse groups. ***p < 0.001, **p < 0.01, *p < 0.05. **e** H&E staining of gastrocnemius muscle of SBMA mice untreated or treated with trehalose (treha) and/or bicalutamide (bical) at 26 weeks of age. Scale bar 50 μm. Head arrow indicate centrally nucleated myofibers. *n* = 4. **f** Analysis of gastrocnemius muscles fiber parameters (perimeter, area, minimum and maximum Feret diameters) in KI AR113Q untreated or treated with treha and/or bical. Each bar represents the mean ± SEM of four independent replicates. ANOVA with a Tukey’s post hoc test among KI AR113Q mouse groups. *p < 0.05. **g** Relative frequency distribution of myofiber min Feret diameter size sorted in KI AR113Q untreated or treated with treha and/or bical. **h** Expression of genes coding for different myosin heavy chain isoforms in SBMA mouse gastrocnemius muscles. RT-qPCRs were performed on total RNA extracted from muscles of untreated or treated with treha and/or bical KI AR113Q mice. Data were normalized to the amount of *Rplp0* mRNA, expressed relative to the levels determined in NTg mice taken as internal reference, and expressed as fold changes. Data are means ± SEM of five independent replicates. ANOVA with Tukey’s post hoc test among KI AR113Q mouse groups. *p < 0.05

Then, we focused our analyses on possible morphological and molecular changes in KI AR113Q mouse skeletal muscle fibers and evaluated possible beneficial effects of bicalutamide and trehalose treatment at the early symptomatic phase (26 weeks of age). Hematoxylin and eosin (H&E) staining (Fig. 4a) of SBMA mouse gastrocnemius showed a higher number of atrophic fibers compared to the NTg mice, accordingly with previous reports in mice and patients [4, 43, 44]. Figure 4b reports the quantification of fiber morphological parameters: perimeter, area, min and max Feret diameters of KI AR113Q muscle fibers were significantly lower than those of NTg animals. Accordingly, the frequency distribution of SBMA muscle fibers’ min Feret diameter revealed a shift toward a smaller fiber size (Fig. 4c). These data indicate a clear muscle pathology at 26 weeks of age. To define the types of fibers present in skeletal muscles of SBMA mice, we assessed the gene expression of different myosin heavy chain (Myh) isoforms. As reported in Fig. 4d, we found an increase of *Myh7* and *Myh1* mRNA, and a decrease in *Myh4* mRNA in SBMA mouse muscle compared to NTg mice confirming the fast-to-slow fiber switching already reported in this mouse model and in patients [44]. The expression of the gene coding for a neonatal isoform of myosin heavy chain (*Myh8*) was also found increased in KI AR113Q mice indicating an attempt of regeneration in their gastrocnemius muscles. This observation is also corroborated by the increase of *Pax7* expression [45]. These parameters remained unmodified by vehicle administration (Fig. 4a–d).

As reported in Fig. 4e, f, trehalose counteracted the effects of ARpolyQ expression increasing the area and the min Feret diameter of the fibers, leading to a shift to the right of the muscle fiber distribution curve (Fig. 4g). The simultaneous treatment with bicalutamide and trehalose augmented the Feret max diameter, and all the treatments tended to increase the perimeter, even if without reaching statistical significance. Regarding the different isoforms of Myh expressed, trehalose strongly diminished *Myh7* gene expression, and all the treatments reduced *Myh1* mRNA, indicating that bicalutamide and trehalose counteract the fast-to-slow fiber switching (Fig. 4h). Furthermore, trehalose reduced *Myh3* mRNA, while both compounds tend to decrease the levels of *Myh8* mRNA but reach statistical significance only with the combined treatment. We did not detect any change in the gene expression of *Myh2* and *Myh4*. Muscle histochemistry of treated KI AR113Q mice confirmed the presence of fibers with a caliber greater than that of untreated mice (Fig. 4e, f).

This set of data suggests that both bicalutamide and trehalose counteract the detrimental effects of ARpolyQ expression in muscle fibers by partially recovering their original morphology, possibly explaining the improvement of the motor behavior observed in treated mice.

### Bicalutamide Reduces ARpolyQ Aggregation and Impacts on the Autophagic Flux in Skeletal Muscle

**Fig. 5:**
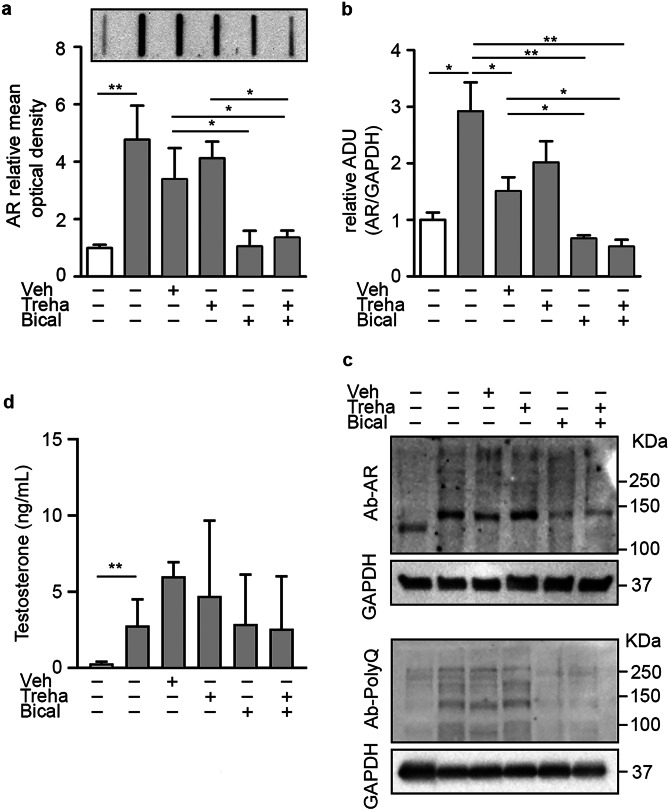
Androgen receptor aggregation and expression in muscle of SBMA mice treated with bicalutamide and/or trehalose. **a** Filter retardation assay in gastrocnemius muscles of non-transgenic (NTg) and KI AR113Q mice to evaluate AR aggregation at 26 weeks of age. White bar, Non-transgenic (NTg); gray bars, KI AR113Q untreated or treated with trehalose (treha) and/or bicalutamide (bical). Inset: representative blot. **b** Densitometric analysis of western blot analysis reporting AR expression in the gastrocnemius muscles of NTg, KI AR113Q and treated KI AR113Q mice at 26 weeks of age. AR levels were normalized using GAPDH as loading control. Each bar represents the mean ± SEM of four independent replicates. Two-tailed Student’s *t*-test between KI AR113Q and NTg mice; ANOVA with Tukey post-hoc test among KI AR113Q mouse groups. *p < 0.05, **p < 0.01. **c** A representative immunoblot of anti-AR antibody staining, and of anti-polyQ antibody staining. **d** Testosterone levels in mouse sera measured by ELISA assay. Each bar represents the mean ± SEM of five independent replicates. **p < 0.01

It is known that, in skeletal muscle, ARpolyQ misfolds, reduces its long-term turnover, aggregates, and perturbs the protein quality control (PQC) system [3, 27, 46]. By FRA, we evaluated the amount of ARpolyQ aggregates in gastrocnemius muscle of KI AR113Q mice. As reported in Fig. 5a, bicalutamide, alone or in combination with trehalose, completely counteracted the formation of ARpolyQ insoluble species typically found in SBMA muscle, while vehicle and trehalose alone did not induce any variation. Then, we analyzed total lysate monomeric ARpolyQ species in western blot (WB) and found that these were 3-fold increased in skeletal muscle of KI AR113Q mice compared to NTg mice (Fig. 5b, c). Importantly, in KI AR113Q mice, bicalutamide was able to reduce the total amount of monomeric ARpolyQ to levels comparable to those found in NTg mice. Unexpectedly, the vehicle for bicalutamide administration also influenced monomeric ARpolyQ even if this effect was lower than that observed in bicalutamide-treated mice. Since polyQ-expanded proteins form micro-aggregates, which can be detected as high molecular weight species, we analyzed these samples also with an antibody raised against the polyQ domain. As reported in Fig. 5c, in KI AR113Q mouse muscle bicalutamide diminished the levels of proteins containing the polyQ tract, while, in this case, no changes were observed in vehicle-treated mice. These results indicate that ARpolyQ aggregation may exert toxic effects in skeletal muscle cells of KI AR113Q mice, even if our colony of mice carries a CAG repeat tract (85 CAG repeats) shorter than the one present in the original colony; these data are also in line with those obtained in the original model [46]. Importantly, bicalutamide increases cytoplasmic ARpolyQ clearance thus reducing its detrimental activity in muscle cells and possibly explaining the beneficial effects of this drug on the motor behavior of KI AR113Q mice. To exclude that the effect of bicalutamide is linked to induced changes in testosterone levels, we measured the amount of this steroid in the mouse sera (Fig. 5d). As expected, the data confirmed that testosterone levels in KI AR113Q mice are higher than in NTg mice, but that these levels are not influenced by both treatments.

**Fig. 6:**
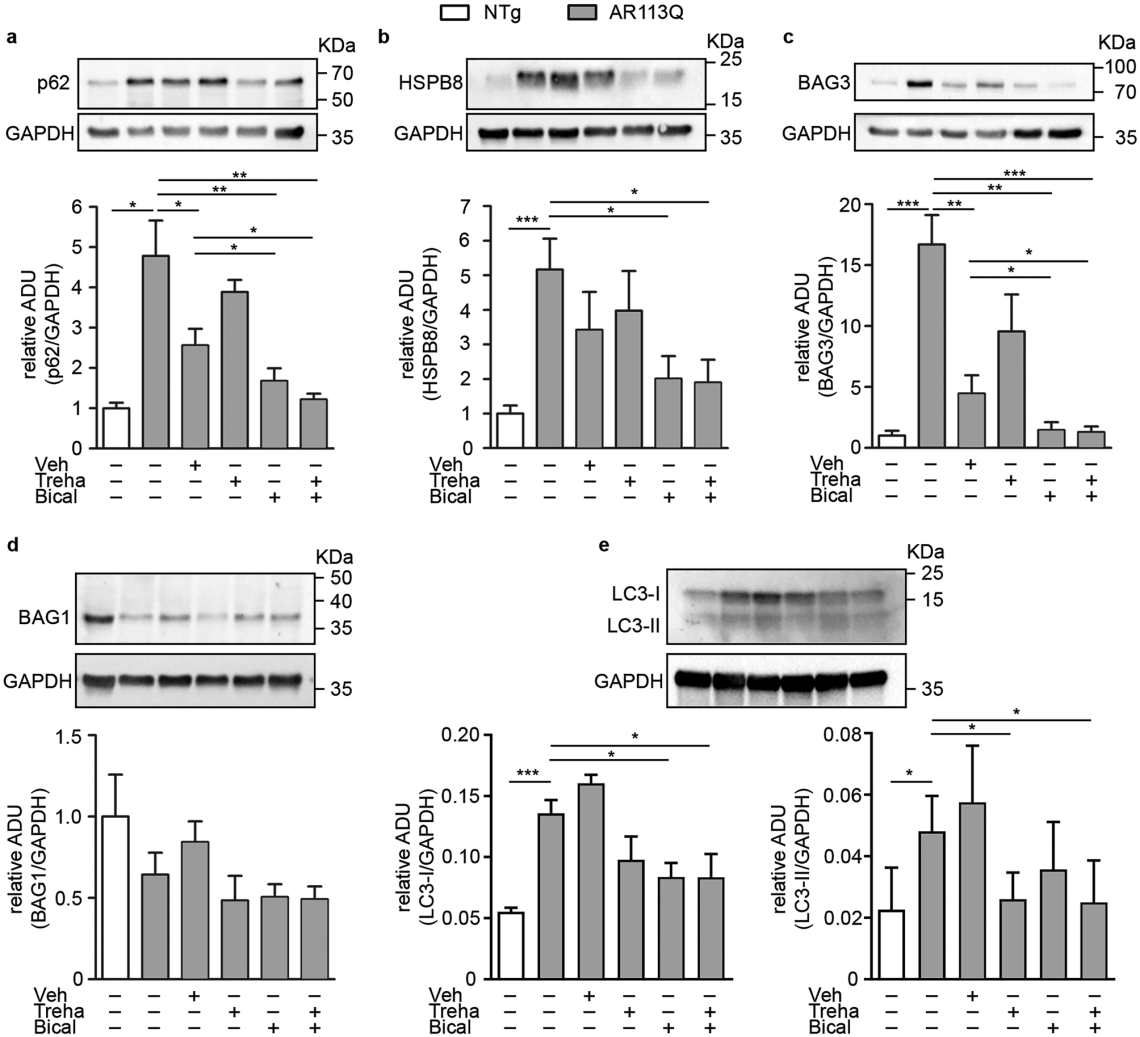
Expression of proteins regulating autophagy and HSPB8-mediated response in muscle of SBMA mice treated with bicalutamide and/or trehalose. Western blot analyses of autophagic markers expression in the gastrocnemius muscles of non-transgenic (NTg), KI AR113Q and treated KI AR113Q mice at 26 weeks of age. **a** SQSTM1/p62; **b** HSPB8; **c** BAG3; **d** BAG1; **e** LC3-I and LC3-II. Each panel reports a representative immunoblot of the specific antibody staining and that of GAPDH used as loading control, and the densitometric analysis of four independent replicates (mean ± SEM). Two-tailed Student’s *t*-test between KI AR113Q and NTg; ANOVA with Tukey’s post hoc test among KI AR113Q mouse groups. *p < 0.05, **p < 0.01***p < 0.001

Evidence supports the important role of autophagy in SBMA [27, 46–48]. We thus evaluated the expression of key proteins involved in autophagy to determine whether bicalutamide and trehalose exert their beneficial effect on SBMA mice influencing the autophagy process. Sequestosome-1 (SQSTM1/p62) recognizes ubiquitinated proteins for their insertion into autophagosomes, and it is considered an autophagy marker. Figure 6a shows the presence of a robust increase of SQSTM1/p62 protein in KI AR113Q mouse gastrocnemius muscle at the early symptomatic stage (26 weeks of age) compared to the same muscle of age-matched NTg mice. Bicalutamide treatment, alone or in combination with trehalose, counteracted the increase of SQSTM1/p62 expression in KI AR113Q mouse gastrocnemius muscle, reducing it to levels similar to control mice. A very similar pattern was also found for the protein levels of HSPB8 (Fig. 6b), which is involved in a peculiar form of autophagy (Chaperone assisted selective autophagy, CASA) essential for the removal of several misfolded proteins causative of NDs, including ARpolyQ, and for muscle maintenance [49, 50]. Of note, HSPB8 acts in a complex with the co-chaperone Bcl-2 Associated Athanogene 3 (BAG3) (Fig. 6c) [51, 52], and the two proteins bind the Heat Shock Protein 70 (HSP70) and the C-terminus of HSC70 interacting protein (CHIP) to form the CASA complex and trigger CASA. HSPB8 and BAG3 also compete with the co-chaperone BAG1 in its association to HSP70-CHIP, a process responsible for proteasomal degradation. We already reported a peculiarity of KI AR113Q mice that have reduced BAG1 levels in muscle, indicating that ARpolyQ protein degradation is preferentially routed to autophagy rather than the proteasome [46]. Unfortunately, we found that neither trehalose nor bicalutamide (and not even the two drugs combined) were able to restore normal BAG1 levels, excluding the involvement of the proteasome in the beneficial activity of these drugs in KI AR113Q mice (Fig. 6d).

Microtubule-associated protein 1A/1B-light chain (LC3-I) is also a well-recognized marker for autophagosomes, to which it associates after its lipidation to LC3-II. We here reported that both LC3-I and LC3-II levels are increased in KI AR113Q mouse muscle and returned to basal levels with treatments (Fig. 6e), indicating a more efficient autophagic flux in the presence of trehalose and/or bicalutamide. At the transcriptional level, LC3 and BAG3 were also increased in KI AR113Q mouse muscle, but their mRNA levels were not influenced by treatments (Supplementary Fig. 4a–e).

These data demonstrate that in early symptomatic muscles mutant ARpolyQ strongly activates the autophagic pathway. Despite this, SQSTM1/p62 accumulates in muscle fibers probably indicating a blockage of the autophagic flux that is solved by bicalutamide treatment.

### Apoptosis of Mutant KI AR113Q Skeletal Muscle Fiber Is Reduced by Bicalutamide and Trehalose

**Fig. 7:**
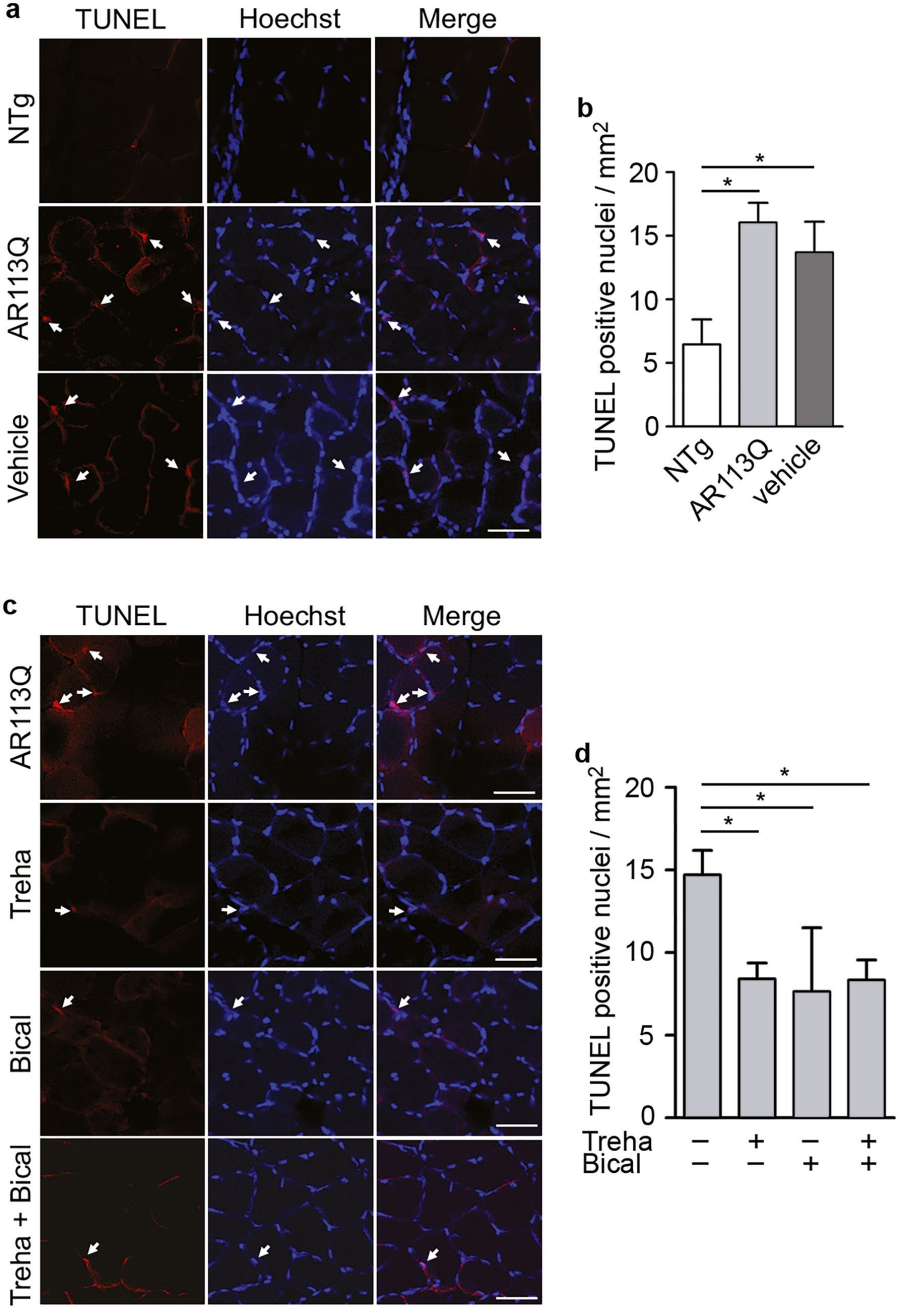
Detection of TUNEL-positive cells in muscles of SBMA mice treated with trehalose and/or bicalutamide. **a** Representative confocal images of TUNEL test performed on gastrocnemius muscle sections from non-transgenic (NTg), KI AR113Q and vehicle (KI AR113Q treated with corn oil, bicalutamide vehicle) mice at 26 weeks of age. Nuclei were stained with Hoechst (blue), while apoptotic nuclei were labeled by TUNEL (red). Hoechst-stained sections and merged images are shown next to the corresponding TUNEL images. White arrows indicate positive nuclei. Scale bar: 50 μ. **b** Quantification of TUNEL-positive nuclei in NTg, KI AR113Q and vehicle mouse muscles. Data are presented as mean ± SEM; *n* = 3–4 mice, each group; ANOVA with Tukey’s post-hoc test. *p < 0.05. **c** Representative confocal images of TUNEL test performed on gastrocnemius muscle sections from KI AR113Q untreated or treated with treha (trehalose) and/or bical (bicalutamide). Nuclei were stained with Hoechst (blue), and apoptotic nuclei were labeled by TUNEL (red). Hoechst-stained sections and merged images are shown next to the corresponding TUNEL images. White arrows indicate positive nuclei. Scale bar: 50 μ. **d** Quantification of TUNEL-positive nuclei in gastrocnemius muscle sections from KI AR113Q untreated or treated with treha and/or bical. Data are presented as mean ± SEM; *n* = 3–6 mice, each group; ANOVA with Tukey’s post hoc test. *p < 0.05

During muscle atrophy apoptosis occurs contributing to the loss of muscle mass [53]. To understand if muscle atrophy detected in KI AR113Q mice involves apoptosis activation, we performed the terminal deoxynucleotidyl transferase-mediated dUTP nick-end labeling (TUNEL) test. The number of apoptotic nuclei was significantly higher in KI AR113Q mouse gastrocnemius muscle compared to NTg mice and did not change following vehicle treatment (Fig. 7a, b). By the analysis of muscle sections from KI AR113Q mice treated either with bicalutamide or trehalose, or with the combination of the two compounds, we found that all the three treatments were able to significantly reduce apoptosis (Fig. 7c, d).

These results reveal the involvement of apoptosis in the pathological processes induced by the mutant ARpolyQ in skeletal muscle and show the capability of bicalutamide and trehalose to prevent apoptosis activation, in line with the improved motor behavior found in KI AR113Q treated mice.

### Trehalose Restores Mitochondrial Complex Levels in Mutant KI AR113Q Skeletal Muscle Fiber

**Fig. 8:**
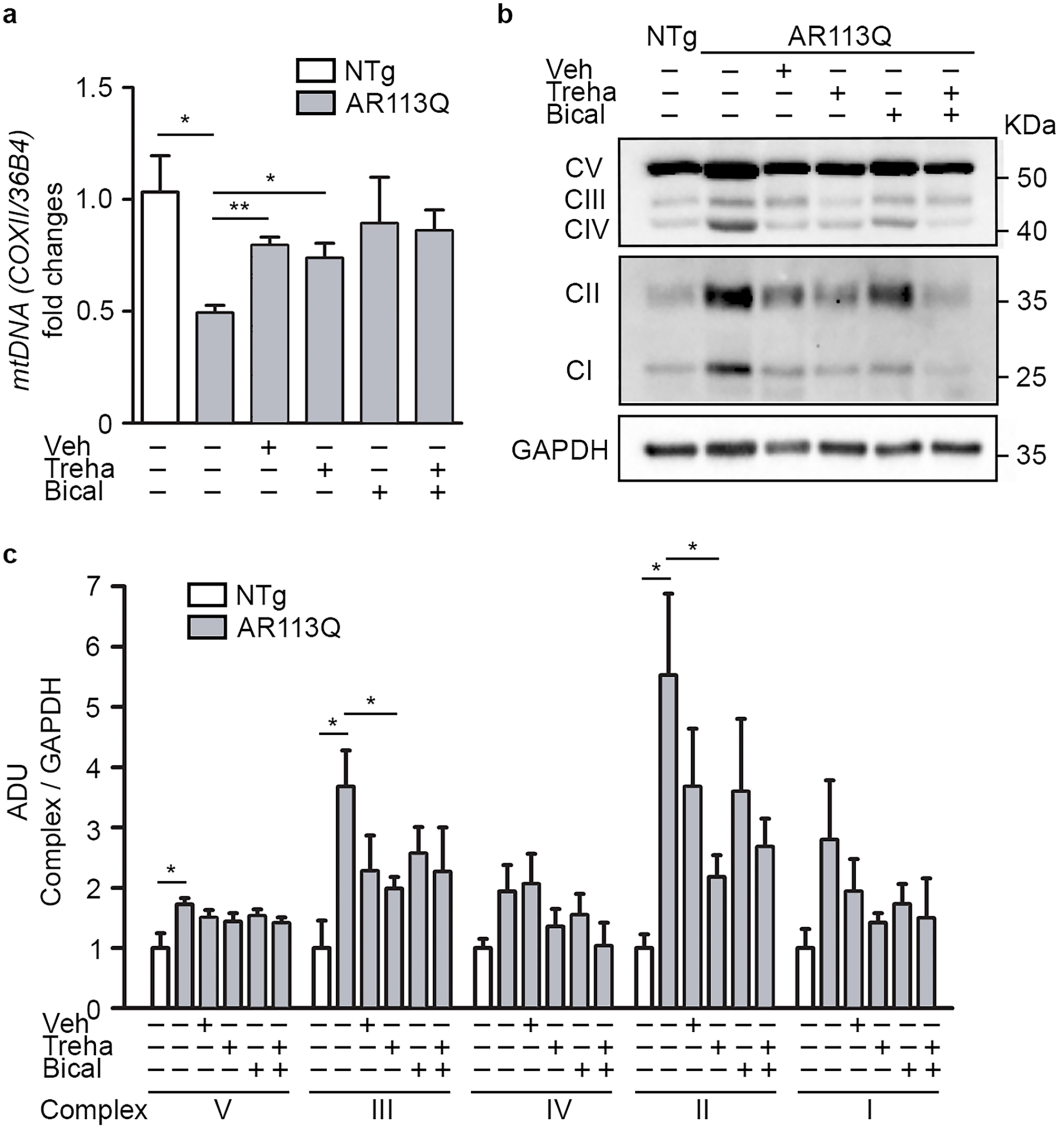
Treatment with bicalutamide and/or trehalose impacts on mitochondria in muscles of SBMA mice. **a** Mitochondrial (mt) DNA content quantification by qPCR as ratio between the expression of the mt gene CoxII and that of the housekeeping nuclear gene *36B4* (*Rplp0*). qPCRs were performed on DNA extracted from muscles of non-transgenic (NTg), KI AR113Q and trehalose (treha)- and/or bicalutamide (bical)-treated KI AR113Q mice. Data are expressed relative to the levels determined in NTg mice taken as internal reference and expressed as fold changes. Each bar represents mean ± SEM of five independent replicates. Two-tailed Student’s *t*-test between NTg and KI AR113Q mice; ANOVA with Tukey’s post hoc test among KI AR113Q mouse groups. *p < 0.05, **p < 0.01. **b** A representative immunoblot of the staining with the anti-OXPHOS complex cocktail antibody staining. Due to the different expression of the complexes, images of the same blot have been reported at separate times of exposure. GAPDH was used as loading control. **c** Densitometric analysis of western blot analysis reporting the expression of different OXPHOS complexes in the gastrocnemius muscles of NTg, KI AR113Q and treated KI AR113Q mice at 26 weeks of age. Each bar represents the mean ± SEM of four independent replicates. Two-tailed Student’s *t*-test between NTg and KI AR113Q mice; ANOVA with Tukey’s post hoc test among KI AR113Q mouse groups. *p < 0.05

It is known that ARpolyQ may exert toxicity also by interfering with mitochondrial functions, and it has been postulated a possible localization of this protein in mitochondria [54]. Mitochondrial abnormalities are also a signature of SBMA disease in the KI AR113Q mouse muscle [44]. We thus evaluated whether mitochondria alterations can be reverted by the administration of bicalutamide and trehalose, that have been proved to improve motor activities in our mouse colony. We analyzed the mtDNA content and the protein levels of OXPHOS complexes in gastrocnemius muscles of control and treated KI AR113Q mice. The levels of mtDNA were evaluated by qPCR as the ratio between the expression of the mitochondrial gene cytochrome c oxidase subunit 2 (*CoxII*) and the housekeeping gene *36B4* (*Rplp0*). As reported in Fig. 8a, muscles of early symptomatic KI AR113Q mice showed a remarkable decrease in mtDNA content indicating mtDNA instability. Trehalose prevented the loss of mtDNA in the muscle of these mice. We noted a strong impact of the vehicle that cannot permit us to evaluate the effect of bicalutamide, administered dissolved in oil. On the contrary, the expression of the electron transport chain complexes is increased in KI AR113Q mouse muscle, possibly indicating an enhanced activity of the intact mitochondria to counteract mtDNA loss (Fig. 8b, c). The levels of the OXPHOS enzymes were decreased by trehalose administration probably reflecting its effect in reducing mtDNA loss (Fig. 8b, c).

These data corroborate an important role of mitochondrial dysfunction already at the early symptomatic stage of the disease and may contribute to explaining the beneficial effect of trehalose against muscle apoptosis or in mediating the improvement of motor function in treated KI AR113Q mice.

## Discussion

Here, we evaluated in vivo the therapeutic potential of bicalutamide and trehalose, administered alone or in combination, in the KI AR113Q mouse model of SBMA. We found that bicalutamide extends the survival of the KI SBMA mice, while both compounds improve mouse motor abilities and partially recover muscle fiber morphology. Bicalutamide also prevented the formation of AR insoluble forms in mouse muscles and possibly restored the autophagic flux. Furthermore, trehalose reverted the mtDNA and the OXPHOS enzymes decrease found in KI AR113Q mice. Finally, we found an activation of the apoptotic process in muscle fibers that was prevented by bicalutamide and trehalose.

We selected trehalose, an autophagy activator, and bicalutamide, a type 1 AR antagonist that reduces the translocation rate of AR from the cytoplasm to the nucleus, with the idea to take advantage of their potential synergistic activities identified in a MN cell model of SBMA [27]. In fact, the cytoplasmic retention of ARpolyQ induced by bicalutamide should enhance its cytoplasmic autophagic clearance stimulated with trehalose, also preventing its potential nuclear toxicity [14, 55]. Unfortunately, this hypothesis was not confirmed in mice, even if, singularly, the two compounds ameliorated different pathological conditions. The KI AR113Q mice was selected as SBMA mouse model, since it well recapitulates the pathological features characterizing the human disease, with ARpolyQ expressed in a physiological manner under the control of its endogenous promoter. Our mouse colony was characterized by a phenotype weaker than that originally described [33], since multiple cycles of breeding reduced the original AR 113Q tract to 85Q; this size is close to the longest elongation of 72Q in AR found in SBMA patients, so far [34]. Therefore, in this mouse colony the AR 85Q well mimicked the aberrant properties of the mutant human ARpolyQ in animal tissues and also retained affected MNs survival in the spinal cord inducing muscle atrophy. Unfortunately, this polyQ contraction resulted in a much longer disease course and increased survival rate that prevented us to obtain the classical survival curve in which more than 50% of the experimental animals die. In addition, we were forced to treat mice measuring alterations in their motor behavior for a very long time (52 weeks). Despite this, we successfully demonstrated that trehalose tended to increase the median survival and bicalutamide increased the percentage of animals surviving after one year.

Bicalutamide and trehalose were initially administered at an early stage of life, before any sign of the disease appeared, i.e., 6 weeks of age. Under these conditions, bicalutamide worsened mouse motor behavior, since in this cohort of animals the onset was anticipated, possibly because the anti-anabolic and antiandrogenic effect of bicalutamide delayed normal muscle development [40]. To overcome this problem, we treated a new group of animals starting at 12 weeks of age (still a pre-symptomatic stage), when muscle development is already completed, monitoring motor behavior till the first two weeks after symptom onset; we found that both bicalutamide and trehalose delayed disease onset in terms of latency to fall on rotarod test, and that bicalutamide also improved grip strength.

Because of the ARpolyQ pathological role, therapies based on AR antagonism could be promising, and other approaches to decrease circulating androgens or to antagonize androgen functions have been tested in SBMA animal models and patients. Dutasteride, a 5alpha-reductase inhibitor that blocks testosterone conversion into its more active metabolite dihydrotestosterone, failed to reduce disease progression in a phase 2 trial, but reduced the number of falls in treated patients [26], a measure that resembles rotarod data found in our study in mice. On the contrary, leuprorelin, a GnRH agonist, diminished nuclear ARpolyQ aggregates and rescued motor dysfunctions in a SBMA mouse model [21], and ameliorated the swallowing function in SBMA patients without any severe adverse effect [20, 23, 56–58]. The non-steroidal AR antagonist flutamide had no effect on the phenotypic expression of SBMA [21], but protected different mouse SBMA models from AR toxicity if combined with treatments lowering androgen production [21, 25]; notably, flutamide suppressed AR transactivation, but it did not inhibit ARpolyQ nuclear translocation that is the main pathogenic mechanism of SBMA. We selected bicalutamide specifically because it is a non-steroidal compound considered as a pure type 1 AR antagonist, that slows down AR-nuclear translocation and AR-mediated transcription and crosses the BBB reaching the spinal cord [59, 60]. Bicalutamide is an FDA-approved and commercially available drug (Casodex®), widely used for long-term administration in prostate cancer therapy, and relatively well tolerated by patients. We found that bicalutamide exerts most of its beneficial effects in muscle rather than in the spinal cord, and reduced ARpolyQ levels and aggregation in muscle, in agreement with data collected in SBMA cells [19, 27]. Trehalose activity has already been demonstrated in many NDs [28–31, 61, 62], but to our knowledge, it has never been tested in in vivo SBMA models. Our findings that trehalose improves SBMA mouse motor performance makes this compound of interest for this type of MN disease. Unfortunately, in humans, orally administered trehalose is partly hydrolyzed by the enterocyte enzyme trehalase into two D-glucose molecules, while a fraction of trehalose is absorbed in the bloodstream [63], as we show by detecting trehalose in the spinal cord after a chronic treatment. Of note, two trehalase-resistant analogs of trehalose (lactulose and melibiose) mimic the beneficial effects of trehalose on ARpolyQ clearance, by stimulating the TFEB-regulated autophagy pathway in SBMA cells [64] thus with potential application in mice and human. Trehalose could also be used for intravenous administration since it is expected to be safe in humans.

A key role of skeletal muscle in SBMA pathogenesis is now clear [44, 45, 65], since patients presents both neurogenic and myopathic changes, with atrophic and grouped fibers, and central nuclei [66], as well as elevated creatine kinase levels compared to individuals affected by other neurogenic diseases [67]. Satellite cells have an impaired fusion process, indicating cell-autonomous ARpolyQ toxicity in muscle [68]. According to published data in human and mouse models [44] reporting glycolytic-to-oxidative fiber-type switch, we found an increased expression of *Myh7* and *Myh1* and a decrease of *Myh4* mRNA. Trehalose, alone or in combination with bicalutamide, counteracted this switch indicating that it neutralizes the harmful effects of ARpolyQ in muscle. Indeed, a reduction in the size of muscle fibers was observed in KI AR113Q mice, with a shift of the distribution toward fibers with a small caliber, while trehalose or bicalutamide led to a partial recovery of the fiber area, and Feret diameters. Also the gene expression of the myosin heavy chain neonatal form (*Myh8*) returned to basal levels after treatments. Since this myosin is re-expressed after an injury, it provides a specific marker of regenerating fibers [69] and its decrease indicates a lower need for renewal by the skeletal muscle of the treated mice, probably due to a minor loss of innervating fibers.

Bicalutamide also counteracts ARpolyQ aggregation and nuclear translocation, in cell models of SBMA [19, 27], and here we found that bicalutamide increases AR turnover in KI AR113Q mice, decreasing AR aggregation measured by FRA in the gastrocnemius muscle, but also counteracting ARpolyQ monomer accumulation in WB assay. It is likely that by binding AR, bicalutamide destabilizes the protein facilitating its degradation. Bicalutamide effect is not linked to changes in testosterone levels, letting us to think that the decrease of AR aggregates is not due to its diminished stimulation. In agreement with reports in human patients [70], our data show that testosterone levels of KI AR113Q mice are in the higher range of normality, indicating that the disease course is not influenced by increased testosterone, as previously reported [71]. The expression of several autophagy genes is enhanced in muscles of KI AR113Q mice, with increased levels of LC3 and of SQSTM1/p62 [46]; similar data were obtained in SBMA patients [43], we also found that SQSTM1/p62 and LC3 protein levels are greatly increased in early symptomatic SBMA muscle, but notably bicalutamide impacts on their accumulation by restoring a proper autophagic activation or flux as supported by the analysis of HSPB8 levels. In fact, HSPB8 is a chaperone induced by harmful events, like proteotoxic stresses, and is increased in different MN diseases to exert its pro-degradative activity on misfolded proteins, like the ARpolyQ [72]. HSPB8 is greatly enhanced in SBMA muscle and bicalutamide restores its basal levels, indicating that bicalutamide prevents ARpolyQ accumulation permitting a normal intracellular autophagic activation.

Also mitochondrial homeostasis is altered by the expression of ARpolyQ (reviewed by [73]), and we found that muscle of KI AR113Q mice presents a reduction in mtDNA copy number (in agreement with previous studies in mice and patients [43, 44, 54, 74]). This reduction caused a compensatory upregulation of respiratory chain complexes that is in line with the shift to a more oxidative fiber phenotype. These data are apparently in conflict with those of Borgia et al. [43] indicating a reduction in the OXPHOS enzyme levels in muscle biopsies of SBMA patients. Nevertheless, we utilized samples derived from mice at early symptomatic disease stage, while human samples from patients are from an overt disease manifestation and years after the onset. Thus, at an early disease stage, muscle fiber tries to fight against ARpolyQ accumulation increasing the activity of the functional mitochondria, a process that probably becomes more and more inefficient with disease progression. Trehalose restored normal levels of mtDNA and partially counteracted OXPHOS complex enhancement, weakening disease phenotype. Hence, the motor behavior improvement could reflect the increased functionality of mitochondria. Bicalutamide recovered mtDNA and OXPHOS levels, but unfortunately in this case an adverse effect of the vehicle precluded us to draw conclusions on the efficacy of the treatment. Notably, high fat diets modify the SBMA disease course interfering in mitochondrial functions [44]; thus, the bicalutamide effects may be due to vehicle accumulation and its impact on mouse metabolism, but it is also possible that corn oil contains molecules with antioxidant activity able to specifically modulate mitochondrial functions.

Therefore, by hampering the nuclear transfer of ARpolyQ and enhancing its turnover, bicalutamide also prevents mitochondrial abnormalities, even if it is unclear whether the decline in mitochondrial function correlates with the decline in mtDNA content and whether there is a causal association with the progressive worsening of the motor behavior.

Changes in mitochondrial structure, stability, mtDNA copy number, and membrane potential might be due to increased excitotoxicity or reactive oxygen species. For example, in skeletal muscle of sporadic cases of amyotrophic lateral sclerosis, another neuromuscular disease, the reduction of respiratory chain complexes activity is associated with diminished mtDNA levels [75], and alterations in mitochondria could be intrinsic to skeletal muscle and not due to denervation [76], a process that generally correlates with an activation of apoptotic pathways [77]. Indeed, apoptosis contributes to denervation-induced muscle atrophy in neuromuscular disorders even if specific molecular mechanisms remain to be further elucidated [78]. We found an increased TUNEL reactivity in KI AR113Q mice at an early symptomatic stage consistent with the observed smaller mean fiber area and diameters, possibly related to reduced presence of toxic signals in muscle. Interestingly, TUNEL signal can also be detected in regenerating muscle fiber leading to hypothesize a role for DNA fragmentation in the process of muscle fiber remodeling in response to toxic stimuli or denervation [79, 80]. Both trehalose and bicalutamide significantly reduced TUNEL reactivity, indicating a reduction in DNA fragmentation.

Our study reveals alterations in muscle morphology and function at an early symptomatic stage of the disease in SBMA KI mice, underlying the importance of developing muscle-targeted therapeutic intervention. Despite bicalutamide and trehalose did not show a synergic effect as we found in MN culture, they counteracted ARpolyQ toxicity with a diminished formation of ARpolyQ aggregates, and a normalization of the autophagic flux in skeletal muscle. Furthermore, bicalutamide and trehalose also impacted mitochondrial activity and prevented apoptosis activation. Altogether, this resulted in a partial recovery of the original muscle fiber morphology and in an improved motor behavior and extended survival of SBMA mice, suggesting that bicalutamide and trehalose may be considered as possible candidates for future clinical trials to be performed on SBMA patients.

## Supplementary Information

Below is the link to the electronic supplementary material.

Supplementary file1 (DOCX 1352 kb)

Supplementary file2 (PDF 1943 kb)

Supplementary file3 (PDF 1874 kb)

Supplementary file4 (PDF 666 kb)

Supplementary file5 (PDF 2104 kb)

Supplementary file6 (PDF 1848 kb)

Supplementary file7 (PDF 2066 kb)

Supplementary file8 (PDF 1640 kb)

Supplementary file9 (PDF 3400 kb)

Supplementary file10 (PDF 1978 kb)

Supplementary file11 (PDF 504 kb)

Supplementary file12 (PDF 24472 kb)

Supplementary file13 (PDF 1644 kb)

Supplementary file14 (PDF 4403 kb)

Supplementary file15 (PDF 2075 kb)

Supplementary file16 (PDF 376 kb)

Supplementary file17 (PDF 4073 kb)

Supplementary file18 (PDF 3622 kb)

Supplementary file19 (PDF 1796 kb)

Supplementary file20 (PDF 1049 kb)

Supplementary file21 (PDF 5181 kb)

Supplementary file22 (PDF 517 kb)

Supplementary file23 (PDF 1862 kb)

Supplementary file24 (PDF 1831 kb)

Supplementary file25 (PDF 678 kb)

Supplementary file26 (PDF 1694 kb)

## Data Availability

The data that support the findings of this study are available from the corresponding authors, upon reasonable request.
