# Supplementary material for: Bicalutamide and Trehalose Ameliorate Spinal and Bulbar Muscular Atrophy Pathology in Mice
Source: Neurotherapeutics. 2023 Jan 30;20(2):524–45. doi: 10.1007/s13311-023-01343-x (PMC10121997; doi:10.1007/s13311-023-01343-x)

**Supplementary Information**

**Supplementary Table 1**

**List of primers used for qPCR reactions**

| mouse *Rplp0*: | 5′-GGTGCCACACTCCATCATCA-3′ (forward) |
| --- | --- |
|  | 5′-AGGCCTTGACCTTTTCAGTAAGT-3′ (reverse) |
| mouse *Myh1*: | 5′- gagggacagttcatcgatagcaa -3′ (forward) |
|  | 5′- gggccaacttgtcatctctca -3′ (reverse) |
| mouse *Myh2*: | 5′- aggcggctgaggagcacgta -3′ (forward) |
|  | 5′- gcggcacaagcagcgttgg -3′ (reverse) |
| mouse *Myh3*: | 5′- GGGACCTTGCCAAGAAGAA-3′ (forward) |
|  | 5′- GTCGTTCCTCACGGTCTTG-3′ (reverse) |
| mouse *Myh4*: | 5′- cacctggacgatgctctcaga -3′ (forward) |
|  | 5′- gctcttgctcggccactct -3′ (reverse) |
| mouse *Myh7*: | 5′- ctcaagctgctcagcaatctattt -3′ (forward) |
|  | 5′- ggagcgcaagtttgtcataag -3′ (reverse) |
| mouse *Myh8*: | 5′- GAGGGCATCCGCATCTG-3′ (forward) |
|  | 5′- GATGAACTGTCCCTCTGGAATAG-3′ (reverse) |
| mouse *Sqstm1/p62*: | 5′- AGGGAACACAGCAAGCT -3′ (forward) |
|  | 5′- GCCAAAGTGTCCATGTTTCA -3′ (reverse) |
| mouse *Hspb8*: | 5′- ATACGTGGAAGTTTCAGGCA -3′ (forward) |
|  | 5′- TCCTTTGACCTAACGCAACC -3′ (reverse) |
| mouse *Lc3*: | 5′- CGTCCTGGACAAGACCA -3′ (forward) |
|  | 5′- CCATTCACCAGGAGGAA -3′ (reverse) |
| mouse *Bag1*: | 5′- GAAACACCGTTGTCAGCACT -3′ (forward) |
|  | 5′- GCTCCACTGTGTCACACTC -3′ (reverse) |
| mouse *Bag3*: | 5′- ATGGACCTGAGCGATCTCA -3′ (forward) |
|  | 5′- CACGGGGATGGGGATGTA -3′ (reverse) |

**Supplementary Fig. 1**

**Bicalutamide and/or trehalose effects on non-transgenic mouse body weight, survival, and motor behavior**

**(a)** Body weight analysis of non-transgenic (NTg) mice (blue, n=13), and NTg mice treated with bicalutamide vehicle (veh, corn oil, purple, n=15), trehalose (treha, green, n=21), bicalutamide (bical, pink, n=8) and trehalose plus bicalutamide (orange, n=12). Treatments started at 6 weeks of age (dashed line) and continued until the sacrifice at 52 weeks of age. Data are expressed as mean ± SEM. **(b)** Kaplan-Meier analysis of survival of NTg mice and NTg mice treated with bicalutamide vehicle, or trehalose, or bicalutamide, or trehalose plus bicalutamide. Colors and number of replicates as in A. **(c)** Rotarod analysis of motor coordination and **(d)** grip strength test for forelimb force in NTg mice and NTg mice treated with bicalutamide vehicle, or trehalose, or bicalutamide, or trehalose plus bicalutamide. Colors and number of replicates as in A. Treatments started at 6 weeks of age (dashed line). Data are expressed as mean ± SEM.


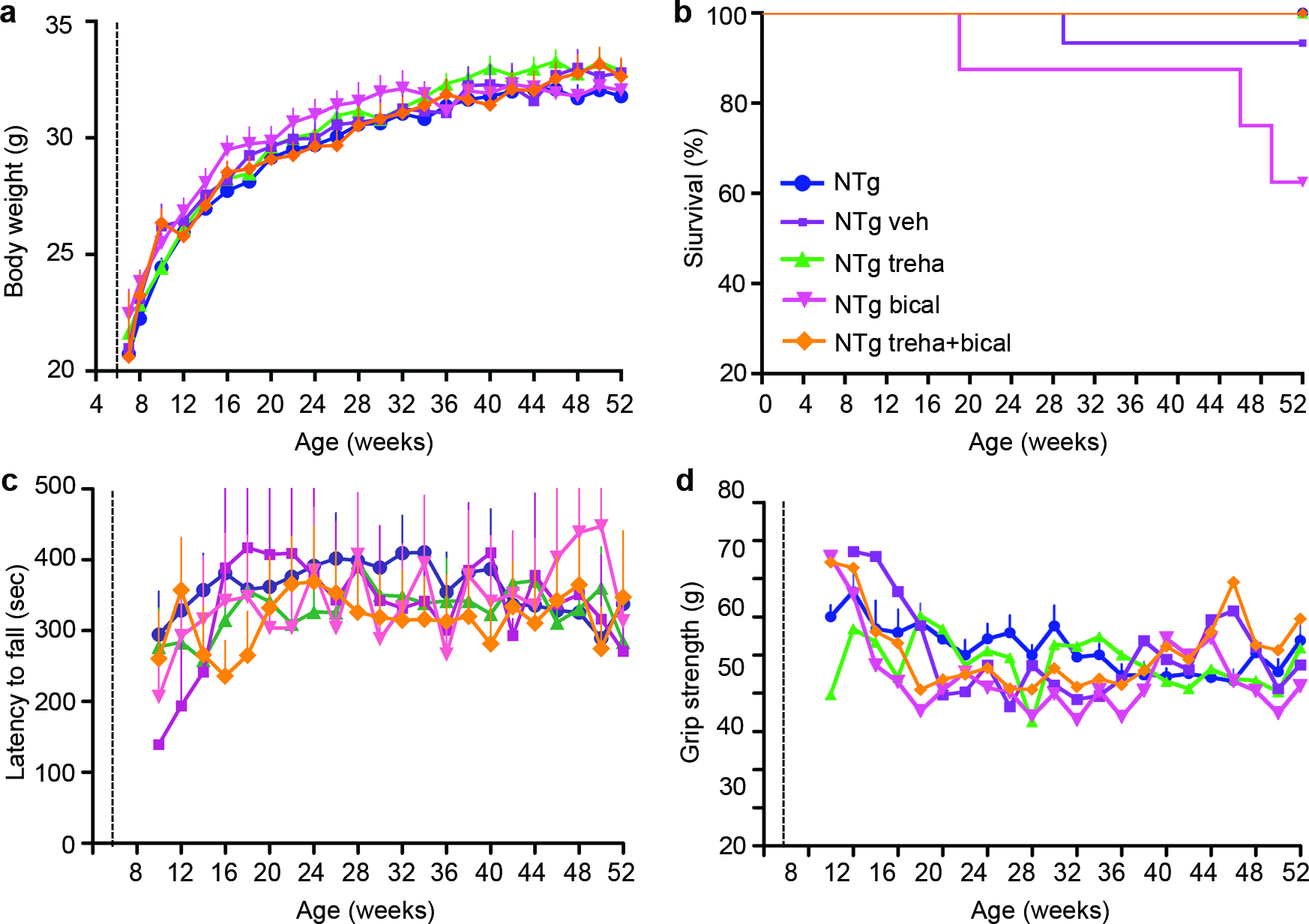


**Supplementary Fig. 2**

**Expression of proteins regulating autophagy and HSPB8-mediated response in the spinal cord of SBMA mice treated with bicalutamide and/or trehalose**

Western blot analyses of autophagic marker expression in the spinal cord of non-transgenic (NTg), KI AR113Q, and treated KI AR113Q mice at 26 weeks of age. **(a)** SQSTM1/p62; **(b)** HSPB8; **(c)** BAG3; **(d)** LC3-II / LC3-II ratio.

Each panel reports a representative immunoblot of the specific antibody staining and that of GAPDH used as loading control, and the densitometric analysis of four independent replicates (mean ± SEM).


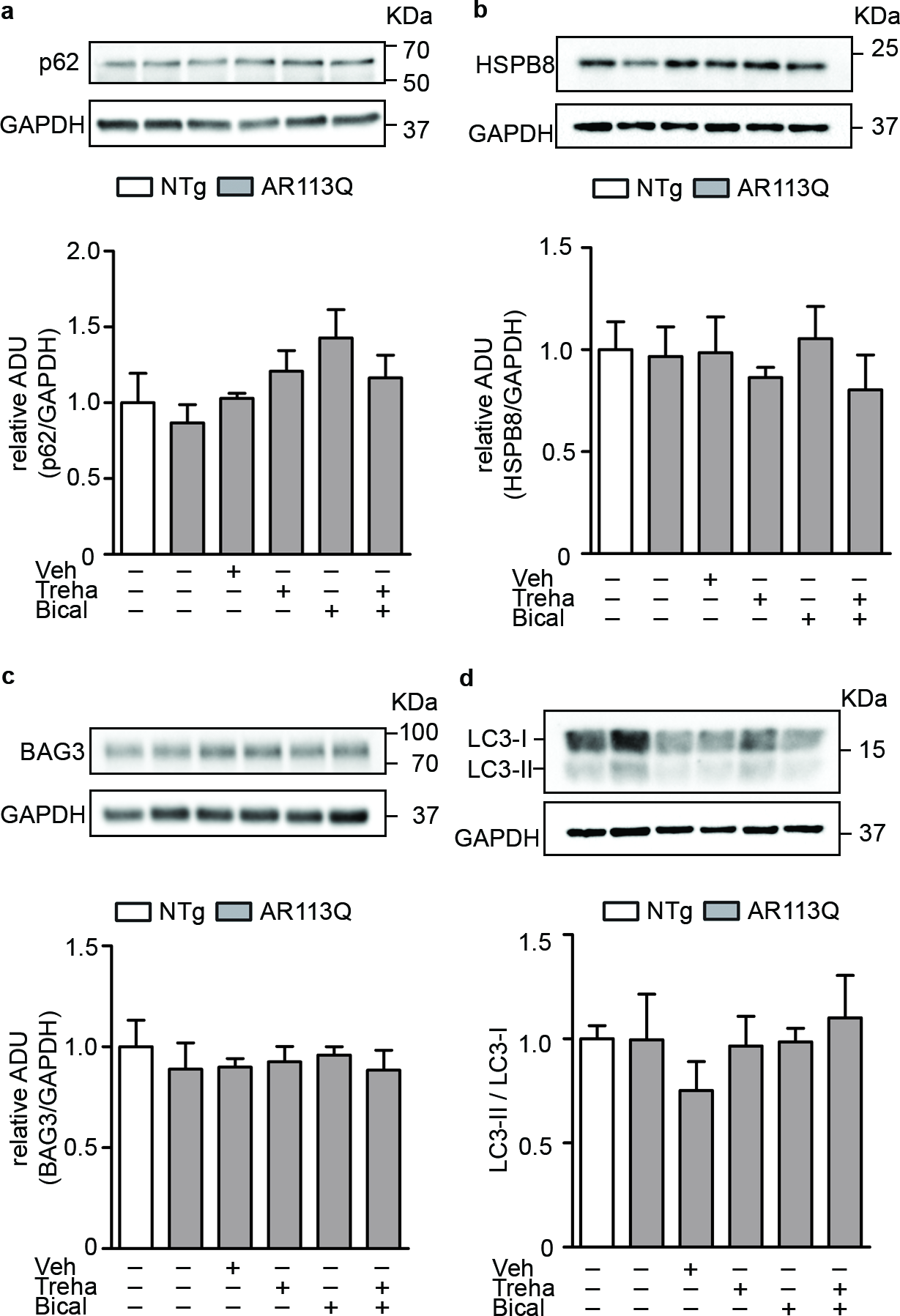


**Supplementary Fig. 3**

**Expression of genes coding for markers of autophagy and of HSPB8-mediated response in the spinal cord of SBMA mice treated with bicalutamide and/or trehalose**

RT-qPCRs were performed on total RNA of NTg, KI AR113Q, and treated KI AR113Q mice at 26 weeks of age. Data were normalized to the amount of *Rplp0* mRNA, expressed relative to the levels determined in NTg mice taken as internal reference, and expressed as fold changes. Data are means ± SEM of 3/5 independent replicates.


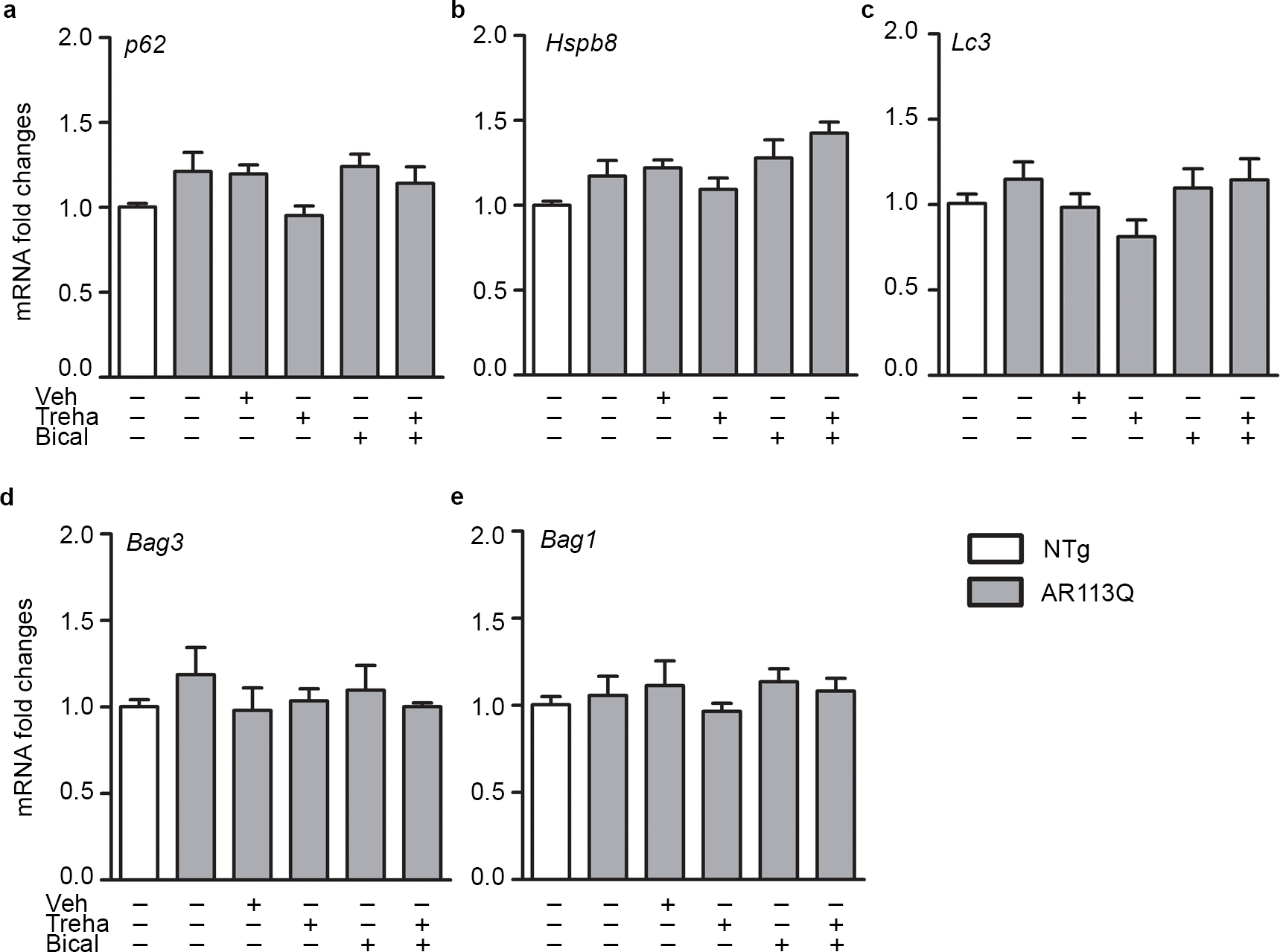


**Supplementary Fig. 4**

**Expression of genes coding for markers of autophagy and of HSPB8-mediated response in the gastrocnemius muscle of SBMA mice treated with bicalutamide and/or trehalose**

RT-qPCRs were performed on total RNA of NTg, KI AR113Q, and treated KI AR113Q mice at 26 weeks of age. Data were normalized to the amount of Rplp0 mRNA, expressed relative to the levels determined in NTg mice taken as internal reference, and expressed as fold changes. Data are means ± SEM of 3/5 independent replicates.


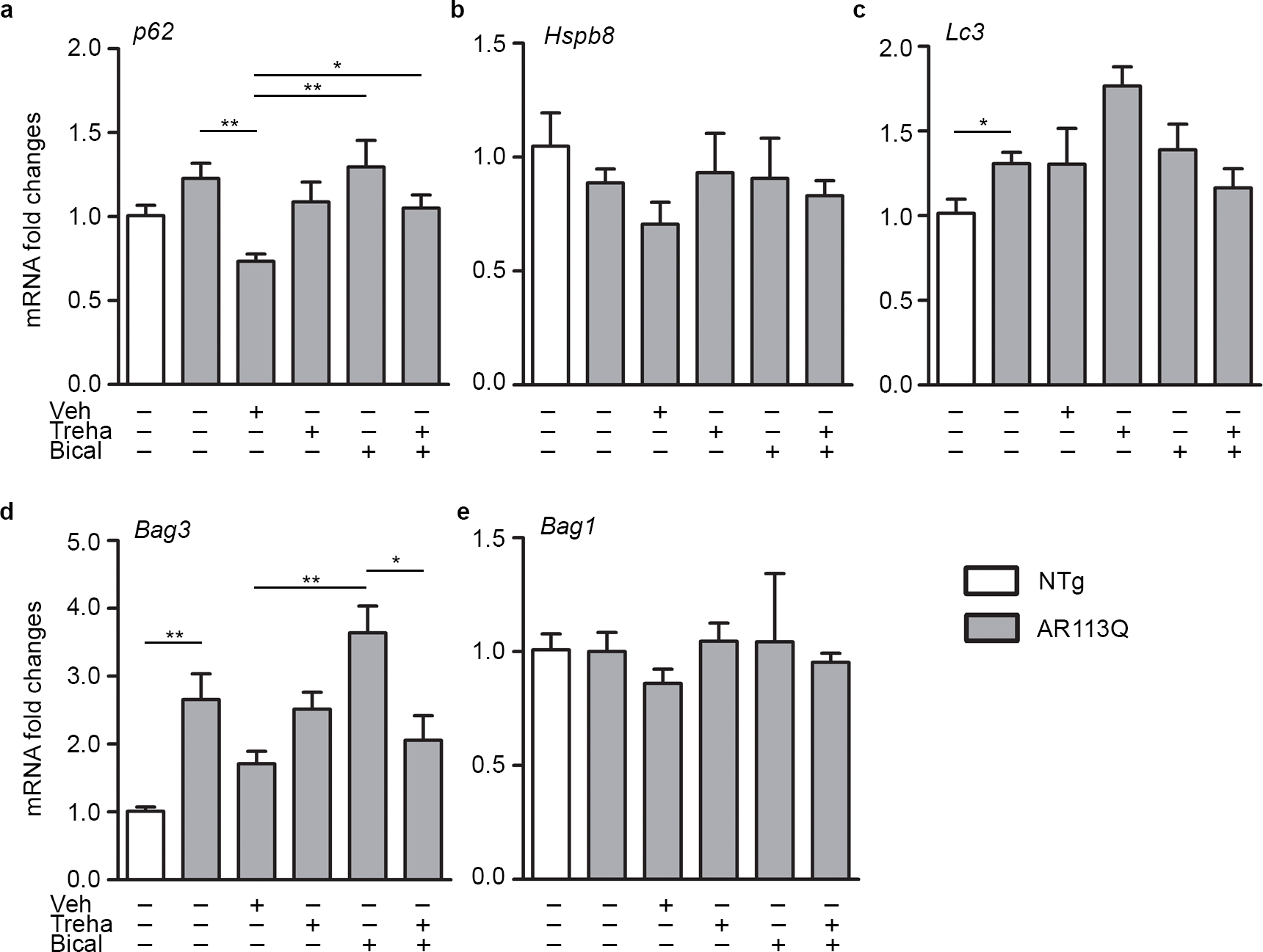

Supplement: Supplementary file 1 — Supplementary file1 (DOCX 1352 kb) [file 13311_2023_1343_MOESM1_ESM.docx]
